# Divisive normalization is an efficient code for multivariate Pareto-distributed environments

**DOI:** 10.1073/pnas.2120581119

**Published:** 2022-09-26

**Authors:** Stefan F. Bucher, Adam M. Brandenburger

**Affiliations:** ^a^Department of Economics, New York University, New York, NY 10012;; ^b^Department of Computer Science, University of Tübingen, 72076 Tübingen, Germany;; ^c^Department of Computational Neuroscience, Max Planck Institute for Biological Cybernetics, 72076 Tübingen, Germany;; ^d^Stern School of Business, New York University, New York, NY 10012;; ^e^Tandon School of Engineering, New York University, Brooklyn, NY 11201;; ^f^New York University Shanghai, Shanghai, China 200122

**Keywords:** divisive normalization, efficient coding, natural stimulus statistics, histogram equalization, Pareto distribution

## Abstract

Divisive normalization is a canonical computation in the brain, observed across neural systems, that is often considered to be an implementation of the efficient coding principle. We provide a theoretical result that makes the conditions under which divisive normalization is an efficient code analytically precise: We show that, in a low-noise regime, encoding an *n*-dimensional stimulus via divisive normalization is efficient if and only if its prevalence in the environment is described by a multivariate Pareto distribution. We generalize this multivariate analog of histogram equalization to allow for arbitrary metabolic costs of the representation, and show how different assumptions on costs are associated with different shapes of the distributions that divisive normalization efficiently encodes. Our result suggests that divisive normalization may have evolved to efficiently represent stimuli with Pareto distributions. We demonstrate that this efficiently encoded distribution is consistent with stylized features of naturalistic stimulus distributions such as their characteristic conditional variance dependence, and we provide empirical evidence suggesting that it may capture the statistics of filter responses to naturalistic images. Our theoretical finding also yields empirically testable predictions across sensory domains on how the divisive normalization parameters should be tuned to features of the input distribution.

## Significance

Divisive normalization is a ubiquitous computation commonly thought to be an implementation of the efficient coding principle. Despite empirical evidence that it reduces statistical redundancy present in naturalistic stimuli, making the relationship between this neural code and the statistics of a stimulus precise has remained elusive. This paper closes this gap by providing a necessary and sufficient condition for divisive normalization to generate an efficient code. The multivariate Pareto distribution found to be efficiently encoded exhibits many stylized features of naturalistic stimulus statistics and provides testable predictions. In an empirical analysis, we find that the Pareto distribution captures the statistics of natural images well, suggesting that divisive normalization may have evolved to efficiently represent stimuli from such distributions.

The brain has to make efficient use of its limited resources to represent and respond to the wide range of stimuli in its environment. An important mechanism by which this can be achieved is divisive normalization (1, 2), which is thought to be a canonical computation in the brain (3). This gain control mechanism (according to which the response of a neuron to its preferred stimulus is suppressed by the intensity of nonpreferred stimuli) permits the representation of potentially unbounded stimuli by biophysically feasible bounded firing rates. Originally proposed for individual neurons in the primary visual cortex (1, 4, 5), this computation has since also been observed at the population level in the primary visual cortex (6–8) and throughout the visual hierarchy (9, 10), as well as in several other neural systems including olfactory pathways (11), the middle temporal area (12, 13), the inferotemporal cortex (14), the hippocampus (15), and in multisensory integration (16). In addition, divisive normalization has been shown to play an important role in value representations (17, 18) and for choice behavior, where it has been proposed to account for violations of the independence of irrelevant alternatives (IIA) axiom of rational choice (19–23; but see refs. 24 and 25). The nonlinear computation has also been suggested to play a role in attentional modulation (12, 26, 27), the modulation of response variability (28), the representation of visual uncertainty (29), and probabilistic inference (30, 31). It is further used in neural network models of the visual system (32, 33) as well as in computer vision and image compression (34).

This ubiquitous array of functions begs the question of what overarching objective the divisive normalization computation achieves. In this paper, we consider this computation’s information-theoretic properties and provide testable conditions for its efficiency that are both simple and general, making them applicable across many of the aforementioned settings.

Since Schwartz and Simoncelli (35) showed empirically that divisive normalization reduces the statistical redundancy present in natural images, a common answer (36) has been that divisive normalization is an implementation of the efficient coding principle (37–41). This principle has been central to our understanding of the visual and other sensory systems (42–44), and it has also provided an account of biases in perception (45) and choice (46–49). Of course, divisive normalization has benefits beyond coding efficiency and redundancy reduction, such as permitting tuning curves that are invariant with respect to “nuisance” dimensions (e.g., maintaining discriminability of orientations regardless of contrast) or ensuring that population responses are easily decodable (e.g., by a linear classifier or winner-take-all competition), among other features (3). Its widespread implementation in the nervous system may thus simultaneously achieve a number of purposes. Here, we focus on the question of whether divisive normalization is indeed an efficient computation, which arises naturally in both the sensory and choice domains.

Despite significant progress (50), an answer to this question in terms of testable conditions for efficiency has remained elusive, since formally relating neural computations to stimulus distributions has proved difficult: “The establishment of a precise quantitative relationship between environmental statistics and neural processing is important … [but] it has been surprisingly difficult to make the link quantitatively precise … [and] specification of a probability distribution over the space of input signals … is a difficult problem in its own right” (ref. 51, p. 1194). We close this gap with a theoretical result that makes precise the conditions on the input distribution under which divisive normalization encodes a stimulus efficiently.

Existing analytical work in the domain of vision has demonstrated that divisive normalization approximately (but not entirely) removes the statistical dependence in models of filter responses to natural images (52–54) such as the conditional normal (35) or lognormal (55) distributions. Moreover, divisive normalization can be viewed as an approximation of the nonlinear radial Gaussianization transformation that removes the statistical dependence of non-Gaussian elliptically symmetric distributions (56), yet divisive normalization itself can do so only imperfectly owing to its bounded range (57, 58). Lyu (50, 59) has quantified the extent to which divisive normalization reduces the statistical dependence of one such elliptical distribution: the multivariate Student’s *t* distribution, which is in the class of Gaussian scale mixture models of natural images (60). He showed that even though divisive normalization approximates the transformation that eliminates this model’s statistical dependencies, it can also increase them in low-dimensional settings.

This literature typically assumes a model of empirical stimulus statistics and derives the predictions of an (approximately) optimal code. It thus represents the first of two common approaches for testing the efficient coding hypothesis (51). The second approach is to examine the statistics of actual neural responses to naturalistic stimuli, in the spirit of Laughlin (40). Here, we pursue instead a third approach that consists of deriving analytically what stimulus distribution a given computation efficiently represents. This is in contrast to Malo and Laparra (54) or Lyu (50), for example, who use similar techniques but who start by assuming a given model of stimulus statistics. Instead, our approach is similar in spirit to that of Ballé et al. (61), who obtain a density model on images by inverting a generalized divisive normalization transform, except that we obtain the input density in analytical closed form. Without making any a priori assumptions about the stimulus statistics, the input distribution we find to be efficiently encoded captures many important features of naturalistic stimulus statistics, as we demonstrate in an analysis of image statistics. Our approach thus provides an additional perspective on the efficiency properties of divisive normalization.

We consider a setting in which an *n*-dimensional input is to be encoded by the divisively normalized firing rates of *n* neurons. The input can be either a stimulus or a representation of a stimulus coming from another neural system upstream. In the context of visual stimuli, the multivariate input could arise, for instance, as the responses of a population of linear filters convolved with the stimulus (35). At least two conditions have to be satisfied for the resulting multivariate representation to be efficient in a low-noise regime. First, it ought to adhere to histogram equalization (40) along each input dimension, which ensures that each output is used equally often. Second, maximizing the Shannon entropy of the output distribution requires—in the absence of constraints—that any statistical dependence across dimensions be removed. We use a formulation of the efficient coding principle that, in a low-noise regime, implies both of these desiderata and thus gives rise to a multivariate analog of the classic criterion of histogram equalization. Specifically, we consider a neural code to be efficient if and only if it maximizes the Shannon mutual information (62, 63) between the *n*-dimensional input and its representation. Since, for sufficiently small noise, this criterion can be approximated arbitrarily well by the requirement that the output distribution is entropy-maximizing, divisive normalization is then efficient whenever it transforms the input distribution into an output distribution that is uniform over the range of values divisive normalization can attain (39, 64).* This allows us to characterize the class of input distributions that are efficiently encoded in a low-noise regime.

We prove that divisive normalization maximizes the entropy of the output distribution if and only if the distribution of inputs in the environment is multivariate Pareto. This suggests that divisive normalization may have evolved as an efficient encoding strategy for heavy-tailed, scale-invariant power-law distributions of the kind that occur in many ecological contexts (66); see also *Discussion*.

The statistical dependence in the multivariate Pareto distribution is also consistent with the conditional variance dependence observed in natural image statistics (35), and it has a representation as a Gamma mixture of independent exponential random variables (providing a link to ref. 60). In an empirical analysis of naturalistic images, we demonstrate that the efficiently encoded Pareto distribution indeed captures the statistics of filter responses to natural images just as well as a common model of natural image statistics does. Divisive normalization may thus be an adaptation, in evolution or development, to various natural contexts with physical quantities whose distributions are characterized by heavy-tailed marginals and an empirically important form of statistical dependence.

We generalize our result by allowing for a representation to come at an arbitrary metabolic cost, which affects the shape of an efficient code (67, 68), and we show how this impacts the optimally encoded input distribution. For example, if costs are linear in the total number of spikes (which constrains the average firing rate), then the entropy-maximizing output distribution is exponential, and the associated input distribution changes accordingly. We provide necessary and sufficient conditions on the stimulus distribution for divisive normalization to be efficient under any member of a large family of cost functions.

Beyond providing a testable prediction on the shape of stimulus distributions that divisive normalization efficiently encodes, our theoretical result also yields empirically testable predictions across sensory domains on how the parameters of the divisive normalization transformation should be tuned to the parameters of the stimulus distribution (35). Specifically, the power index in the normalization function matches the shape parameter of the stimulus distribution, while the normalization weights are the inverses of the scale parameters of the stimulus distribution. Our theoretical predictions thus open the door to systematic experimental tests of the efficiency properties of empirically observed divisive normalization.

## Results

We consider a multivariate stimulus (or input from another neural system) modeled as an *n*-dimensional random vector $S=(S_{1},\ldots ,S_{n})$ taking values $s\in R^{n}$, where, for all *i*, $s_{i}>\mu_{i}$ for some $\mu_{i}\in R$. The distribution of this stimulus is described by a continuous probability density function (pdf) $f_{S}$. The support of this density is semi-infinite, bounded below by the constant $\mu \in R^{n}$, so that shifting the stimulus ***s*** by μ gives rise to the positive input x=s−μ. This input is encoded by a population of *n* neurons whose (mean) firing rates are given by the divisive normalization function (3)

[1] $$r_{i}(x)=\gamma \frac{x_{i}^{\alpha }}{b^{\alpha }+\sum_{j=1}^{n}\lambda_{j}x_{j}^{\alpha }},\;\;i=1,\ldots ,n,$$

for x≥0 and finite parameters γ>0, α>0, *b* > 0, and $\lambda_{j}>0$. The encoding $r:R_{+}^{n}\to R_{+}^{n}$ gives rise to an *n*-dimensional representation. The parameter *γ* is often interpreted as the maximal firing rate, the parameter *b* as a semisaturation constant, and *λ_j_* are normalization weights. One possible interpretation of *α* in the context of vision neuroscience is that $x_{i}^{\alpha }$ models the activation resulting from a linear filter response that is then normalized. While numerous functional forms have been proposed to describe divisive normalization (59, 69), our formulation generalizes the normalization equation in Carandini and Heeger (ref. 3, equation **10**) to include different weights *λ_j_* in the normalization pool. For analytical tractability, we assume that these normalization weights can differ across input dimensions but not across output dimensions.

### Constraints and Metabolic Costs.

To assess the efficiency of the divisive normalization transform, we have to specify the class of permissible codes with which to compare it, as well as the efficiency criterion. We start by defining the former. Specifically, we compare divisive normalization with any encoding $g:R^{n}\to \Delta$ whose codomain is restricted to

[2] $$\Delta \equiv \left\{y\in R_{+}^{n}:\sum_{i=1}^{n}\lambda_{i}y_{i}<\gamma \right\}$$

so that the output y=g(x) resulting from any feasible input ***x*** respects an upper bound *γ* on the λ-weighted sum of firing rates ***y***. In addition to this constraint, we allow for a metabolic cost of a representation (70–72). We assume that a representation $y\in R_{+}^{n}$ comes at a cost c(y), where *c* is an arbitrary cost function that is continuous and bounded on Δ.

The constraint is motivated by the fact that the range of values attained by divisive normalization is bounded by a linear constraint. The following proposition shows that Δ is exactly the range of values the divisive normalization function (Eq. **1**) attains:

Proposition 1. *The divisive normalization function* ***r*** *is invertible and its image is given by the simplex* Δ.

The proof of *Proposition 1* is based on an application of the matrix determinant lemma (e.g., ref. 73, theorem 18.1.1); it is given in *SI Appendix* along with all other proofs. Note that the bound *γ* also implies an upper bound $\gamma /\lambda_{i}$ on the firing rate of each individual neuron, but not all neurons can simultaneously spike at their maximal firing rate. We take this constraint, which may arise from physiological limitations, as given and thus evaluate the efficiency of divisive normalization relative to the class of encodings that respect this same upper bound on the (weighted) sum of (nonnegative) firing rates.

Note that a bounded codomain is not only empirically plausible, but also mathematically necessary for considering “lossy compression,” since an unbounded range of firing rates amounts to perfect coding capacity. Moreover, efficiency among all encodings respecting the constraint Δ is a necessary condition for efficiency among an even larger class of bounded representations with which divisive normalization could potentially be compared, because divisive normalization does adhere to the constraint Δ. Whether neural codes with image Δ are efficient even among those with more general representations is beyond the scope of this paper, but an interesting question for future research. Imposing suitable additional structure on the metabolic cost function, for example, would likely result in such a constraint.

### Efficiency Criterion.

To state the conditions under which a neural code is efficient, we employ a formalization of the efficient coding hypothesis whose criterion is based on the mutual information between the input ***X*** and its noisy representation

$$\tilde{Y}=g(X)+\varepsilon ,$$

where the additive noise ε is a random vector in $R^{n}$ and is independent of ***X***. (The divisive normalization function ***r*** is one example of an encoding ***g***, so that the noise applies to the output of the transformation.)

We assume a low-noise regime in which the entropy h(ε) of the noise is sufficiently small that the mutual information between the input and its noisy representation can be approximated by the entropy of the distribution of outputs Y=g(X).

Proposition 2. *Given independent random vectors* ***X*** *and* ε *in* $R^{n}$ *and a map* $g:R^{n}\to R^{n}$, *the mutual information* I(X;g(X)+ε) *can be approximated arbitrarily closely by the entropy* h(g(X)), *given sufficiently small entropy* h(ε).

According to this result, the efficient coding hypothesis amounts to maximization of the entropy of the output distribution (74, 75) net of metabolic costs.^†^ We thus consider a stimulus encoding $g:R^{n}\to \Delta$ to be efficient if the resulting output distribution maximizes, among all distributions with support equal to Δ, the entropy of ***Y*** net of the expected cost E[c(Y)].

### Characterizing the Optimal Output Distribution.

Ignoring metabolic costs, our efficiency criterion amounts to a multivariate version of histogram equalization and requires that all feasible outputs occur equally often. Among all distributions attaining the same range of values as divisive normalization, the entropy-maximizing distribution of mean firing rates is uniform over its simplex support Δ. Note that, constrained to such a support, the uniform distribution is not statistically independent across dimensions (as would be the case in absence of constraints), yet this distribution does result from the optimal encoding.

The following proposition (cf. 76) characterizes the optimal output distribution taking into account any metabolic costs, allowing for potentially more empirically realistic firing-rate distributions.

Proposition 3. *Fix a random vector* ***Y*** (*the representation*) *with bounded support* C *in* $R_{+}^{n}$ *and a continuous and bounded cost function* $c:C\to R_{+}$*. The pdf of the distribution that maximizes the entropy of* ***Y*** *net of the expected cost*, *i.e.*, *that maximizes* h(Y)−E[c(Y)], *among all distributions with support* C, *is given by*

[3] $$f_{Y}(y)=\frac{e^{-c(y)}}{\int_{C}e^{-c(z)}dz}$$

*for* ***y*** *in* C.

For example, assuming that the cost of a representation is linear in the sum of all firing rates gives rise to a truncated exponential output distribution $f_{Y}(y)\propto 1_{\left\{y\in C\right\}}\exp\left(-\kappa \sum_{i=1}^{n}y_{i}\right)$, which is in line with empirically observed firing rates in response to natural scenes (77, 78). Using high firing rates less frequently, it optimally balances the informational benefit of a wide range of firing rates with the cost of relying on metabolically costly high firing rates. Other special cases of interest include costs that take the quadratic form c(y)=κy·y, in which case $f_{Y}(y)$ is truncated normal on C, and costs that are constant in ***y***, in which case $f_{Y}(y)$ is uniform on C.

### Characterizing the Efficiently Encoded Input Distribution.

By mapping inputs ***x*** to their representations r(x), divisive normalization transforms any stimulus distribution $f_{S}$ into a corresponding distribution of outputs or firing rates. Our main mathematical result describes this transformation by relating any stimulus distribution to the output distribution that results under divisive normalization and vice versa.

Theorem 1. *Let* ***X*** *in* $R_{+}^{n}$ *be a random vector with continuous pdf* $f_{X}$ *and* $r:R_{+}^{n}\to \Delta$ *be the divisive normalization mapping. Let* Y=r(X) *and* $y=(y_{1},\ldots ,y_{n})=r(x)$ *denote the output of the divisive normalization transformation*, *with pdf* $f_{Y}$*. Then the two pdfs satisfy*, *for any* $x\in R_{++}^{n}$,

[4] $$f_{X}(x)=\gamma^{n}\alpha^{n}\frac{b^{\alpha }\prod_{i=1}^{n}x_{i}^{\alpha -1}}{{\left(b^{\alpha }+\sum_{i=1}^{n}\lambda_{i}x_{i}^{\alpha }\right)}^{n+1}}\times f_{Y}(y).$$

The proof uses the change-of-variables formula for random vectors (e.g., ref. 79, theorem 8.1.7) and again relies on the matrix determinant lemma to compute the determinant of the Jacobian of ***r*** in closed analytical form.

Given *Proposition 2* and the shape of the optimal output distribution provided by *Proposition 3*, *Theorem 1* lets us characterize, for any metabolic cost function, the stimulus distribution for which divisive normalization is an efficient encoding in a low-noise regime.

**Fig. 1.**
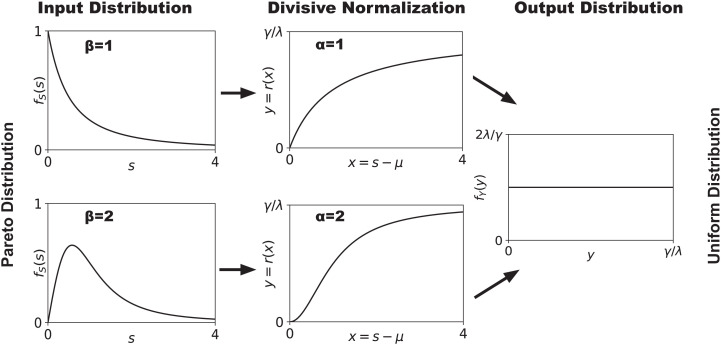
Univariate example of how divisive normalization transforms a stimulus distribution into a distribution over outputs. If a stimulus *s* follows a Pareto distribution (*Left* column), with shape parameter *β* (and *μ* = 0, *σ* = 1), the divisive normalization transform of *x* = *s* (*Center* column), with appropriately chosen parameter *α*, ensures that the resulting representation y=r(x) follows the entropy-maximizing uniform distribution (*Right* column).

Fig. 1 illustrates this for the univariate special case (*n* = 1) absent metabolic costs. Histogram equalization on [0,γ/λ] requires that the output distribution $f_{Y}(y)$ is uniform over this range. A univariate Pareto distribution with pdf $f_{X}(x)=\beta x^{\beta -1}/{\left(1+x^{\beta }\right)}^{2}$ is thus mapped into an entropy-maximizing uniform distribution by a transformation whose derivative is $\gamma \alpha \lambda x^{\alpha -1}/{\left(\lambda (1+x^{\alpha })\right)}^{2}$ as in *Theorem 1* (with α=β and $\lambda =b^{\alpha }$), which indeed integrates to the univariate divisive normalization formula $\gamma x^{\alpha }/(b^{\alpha }+\lambda x^{\alpha })$.

For the general case, the following result states the condition on the stimulus distribution for divisive normalization to maximize, among all distributions with support equal to Δ, the entropy of the resulting output distribution net of expected costs:

Theorem 2. *Divisive normalization of* x=s−μ *is an efficient encoding of a stimulus* ***S*** *for a cost function c if and only if* 1) *the stimulus distribution has joint pdf*, *for* s>μ,

[5] $$\begin{array}{l} f_{S}(s_{1},\ldots ,s_{n};\mu ,\sigma ,\beta ,\gamma ,b,c)\\ \;=\gamma^{n}\beta^{n}\frac{\prod_{i=1}^{n}{\left(s_{i}-\mu_{i}\right)}^{\beta -1}/b^{\beta }}{{\left(1+\sum_{i=1}^{n}{\left(\frac{s_{i}-\mu_{i}}{\sigma_{i}}\right)}^{\beta }\right)}^{n+1}}\\ \;\;\times \frac{e^{-c(r(s-\mu ))}}{\int_{\Delta }e^{-c(z)}dz}, \end{array}$$

*and* 2) *the parameters satisfy* α=β *and* $\lambda_{i}={\left(b/\sigma_{i}\right)}^{\alpha }$.

Note that the efficiently encoded distribution depends on the metabolic cost function *c* and also on the parameters of the divisive normalization transform. The efficiently encoded input distribution shares the parameters *γ* and *b* with the divisive normalization transform. Additionally, efficiency requires that the exponent *α* of the divisive normalization transform matches the shape parameter β>0 of the input distribution and that the normalization weights *λ_i_* are inversely proportional to the scale parameters $\sigma_{i}>0$. This reflects the fact that scaling inputs requires adjusting normalization weights to maintain the efficiency condition of *Theorem 1*. Of course, other parameterizations of this distribution are possible, but the scale parameters *σ_i_* reflect the extent to which *λ_i_* and *b* are exchangeable, and they will be helpful below.

**Fig. 2.**
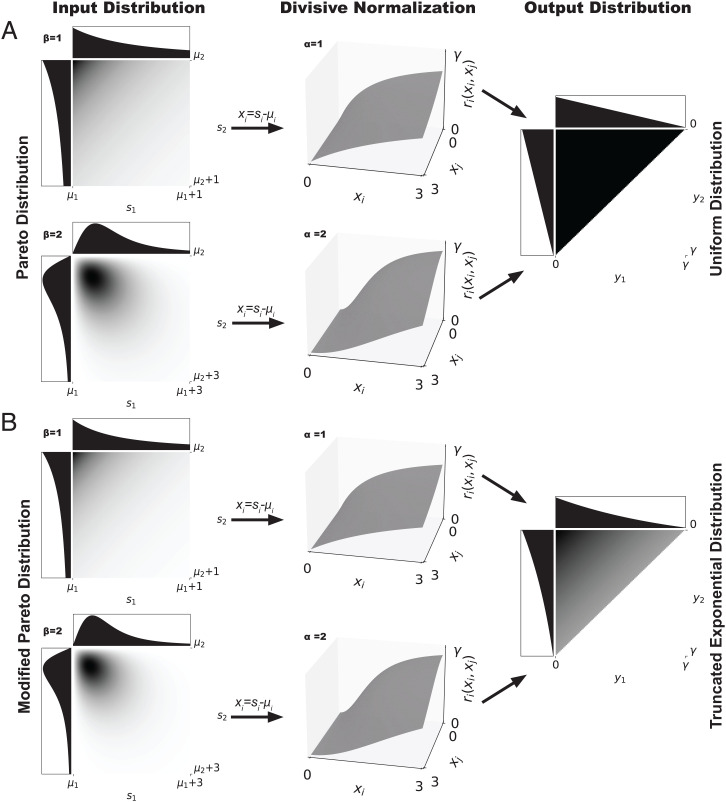
Divisive normalization transforms the distribution of stimulus ***s*** into a distribution over outputs y=r(x). In this bivariate example with σ=λ=1, *b* = 1, and two values of *α* and *β*, joint probability densities are plotted with darker color representing higher density. Marginal densities are shown in the adjacent plots. The Pareto distributions in *A* are transformed into the uniform distribution over the simplex Δ (black triangle) that is efficient absent metabolic costs (*Theorem 3*). *B* shows the qualitatively similar stimulus distributions that are transformed into the truncated exponential distribution that is efficient under linear metabolic costs (*Theorem 2*).

Fig. 2 illustrates *Theorem 2* for the bivariate case and metabolic costs that are constant (Fig. 2*A*) or linear (Fig. 2*B*) in the sum of the firing rates of all neurons. Note that the input distribution that divisive normalization efficiently encodes has a higher density for small values for linear metabolic costs than it does absent metabolic costs. This reflects the fact that, in the presence of metabolic costs, higher firing rates should be used less frequently.

### Constant Metabolic Costs.

An important special case of interest is constant metabolic costs, since the problem then reduces to maximizing information transmission, which is a common formulation of the efficient coding hypothesis. *Theorem 3* states the result for this special case, which makes a particularly sharp prediction that can be viewed as a benchmark generalized by *Theorem 2*. Moreover, Fig. 2 demonstrates that the qualitative shape of the efficiently encoded stimulus distribution appears to be robust to the exact assumption on metabolic costs. Recall that the joint survival (or complementary cumulative distribution) function ${\bar{F}}_{S}(s)$ of a random vector ***S*** is defined as

$${\bar{F}}_{S}(s)=P_{S}(S_{1}>s_{1},\ldots ,S_{n}>s_{n}).$$

Theorem 3. *Divisive normalization of* x=s−μ *is an efficient encoding of a stimulus* ***S*** *under constant metabolic costs if and only if* 1) *the stimulus distribution is a multivariate Pareto type III distribution with joint survival function*

[6] $${\bar{F}}_{S}(s_{1},\ldots ,s_{n};\mu ,\sigma ,\beta )={\left[1+\sum_{i=1}^{n}{\left(\frac{s_{i}-\mu_{i}}{\sigma_{i}}\right)}^{\beta }\right]}^{-1}$$

*and joint pdf*

[7] $$f_{S}\left(s_{1},\ldots ,s_{n};\mu ,\sigma ,\beta \right)=\beta^{n}\frac{n!\prod_{i=1}^{n}\frac{1}{\sigma_{i}}{\left(\frac{s_{i}-\mu_{i}}{\sigma_{i}}\right)}^{\beta -1}}{{\left(1+\sum_{i=1}^{n}{\left(\frac{s_{i}-\mu_{i}}{\sigma_{i}}\right)}^{\beta }\right)}^{n+1}}$$

*for* s>μ, *and* 2) *the parameters satisfy* α=β *and* $\lambda_{i}={\left(b/\sigma_{i}\right)}^{\alpha }$.

*Theorem 3* is a corollary of *Theorem 2* for the special case of constant (or zero) metabolic costs. According to *Theorem 3*, which is illustrated for the bivariate case in Fig. 2*A*, the efficiently encoded stimulus distribution is a particular multivariate Pareto type III distribution (80, 81).^‡^ Note that this particular Pareto type III distribution is parameterized by a homogeneous shape parameter β>0 along with location parameters *μ_i_* and scale parameters $\sigma_{i}>0$. As above, efficiency requires that the parameters *λ_i_* and *α* of the normalization formula should be tuned to the stimulus distribution as follows: The exponent *α* should be set to the shape parameter *β* of the distribution, and the normalization weights $\lambda_{i}={\left(b/\sigma_{i}\right)}^{\alpha }$ should be inversely proportional to the (transformed) scales of the distribution. Note that Eq. **7** is obtained from Eq. **5** by imposing constant metabolic costs and evaluating the resulting integral in the denominator, which results in the cancellation of *γ* and *b* (see proof in *SI Appendix*). Under constant metabolic costs this leaves *γ* and *b* as free parameters. As mentioned above, the parameter *γ* can be interpreted as the constraint on the sum of firing rates, and *b* is a semisaturation constant.

The joint and conditional densities of this Pareto type III distribution are plotted in *SI Appendix* for a range of parameter values. Its marginal distribution is a univariate Pareto type III with cumulative distribution function (cdf)

[8] $$F_{S_{i}}\left(s_{i};\mu_{i},\sigma_{i},\beta \right)=\frac{1}{1+{\left(\frac{s_{i}-\mu_{i}}{\sigma_{i}}\right)}^{-\beta }}$$

for $s_{i}>\mu_{i}$, whose mean is, for β>1, given by

[9] $$E[S_{i}]=\mu_{i}+\sigma_{i}\frac{\pi /\beta }{\sin(\pi /\beta )}.$$

*SI Appendix* contains expressions for the variance and further moments. The marginal Pareto distribution is heavy tailed and for $\mu_{i}=\sigma_{i}=\beta =1$ it is an exact power law

[10] $$F_{S_{i}}(s_{i};\mu_{i}=1,\sigma_{i}=1,\beta =1)=1-1/s_{i}$$

for $s_{i}\in [1,\infty )$. This observation is interesting given that many naturally occurring quantities exhibit approximate power-law characteristics (82–84). For $\mu_{i}=0$, the marginal distribution is a univariate log-logistic distribution, sometimes also referred to as a Fisk (85) distribution.

### Empirical Analysis of Natural Stimulus Statistics.

If divisive normalization has evolved as an efficient computation, our result suggests that it may have adapted to environments whose stimulus statistics are well described by multivariate Pareto (type III) distributions. We examine this hypothesis in the visual domain where there is considerable empirical evidence on stimulus statistics to which we can relate our result. In particular, we ask whether the Pareto distribution we have found to be efficiently encoded exhibits the kind of statistical dependence that is commonly found in filter responses to natural images (35, 51, 56, 86). We first note that the pairwise covariance of the efficiently encoded Pareto distribution (Eq. **6**) is, for β>2 and i≠j, given by ref. 80, equation 6.1.29:

[11] $$\begin{array}{ll} & \text{Cov}(S_{i},S_{j})\\ & \;=\sigma_{i}\sigma_{j}{\left(\Gamma \left(\frac{\beta +1}{\beta }\right)\right)}^{2}\left(\Gamma \left(\frac{\beta -2}{\beta }\right)-{\left(\Gamma \left(\frac{\beta -1}{\beta }\right)\right)}^{2}\right), \end{array}$$

where Γ is the gamma function.

**Fig. 3.**
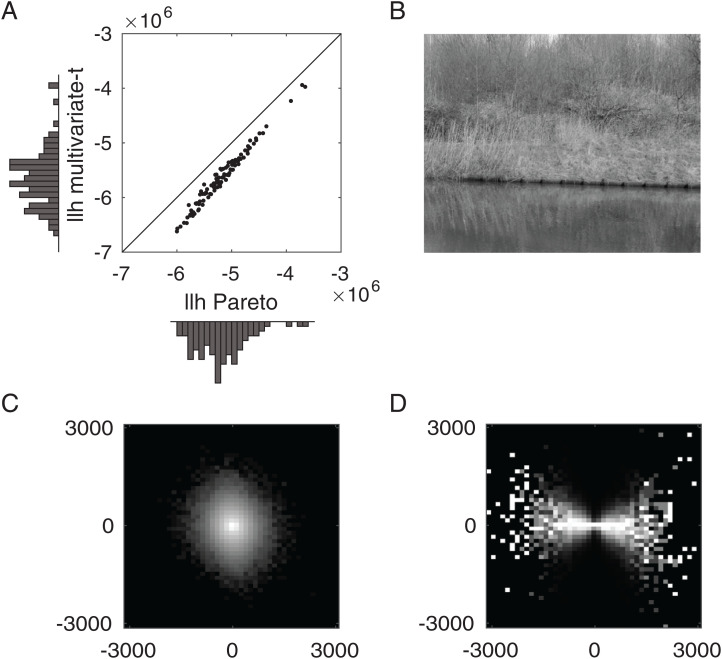
Fitting natural stimulus statistics (see *Materials and Methods* for details). (*A*) Scatterplot showing the log-likelihood (llh) of the best-fitting Pareto (*x* axis) and multivariate-*t* (*y* axis) models to the statistics of naturalistic images from the van Hateren dataset (87). Each dot corresponds to one image and the histograms show the corresponding marginal distributions. (*B*) An (additional) example image from the van Hateren dataset, with log-transformed brightness for better visibility. (*C*) Joint histogram for the example image of the responses of two filters with orientations of 45^∘^ and 135^∘^ counterclockwise, respectively (brightness on log scale). (*D*) Corresponding conditional histogram of the same filter responses, showing the distribution of responses of one filter (*y* axis) conditional on the response of the other filter (*x* axis). The brightness is rescaled in each column to use the full range of intensities, in line with the literature (35).

To assess whether the Pareto distribution and its correlation structure are an empirically relevant model of natural image statistics, we performed a simple exploratory analysis (Fig. 3 *A–D*) using images from the van Hateren image dataset (87). We obtained the joint histogram of the responses of a pair of filters differing in orientation (shown in Fig. 3*C* for the example image of Fig. 3*B*), as well as the corresponding conditional histogram (Fig. 3*D*). The characteristic “bow-tie” shape of the conditional histogram is an empirical regularity observed in numerous naturalistic stimuli (35, 86).

Using the filter response data, we obtained maximum-likelihood estimates, for each image separately, of the parameters of a bivariate Pareto distribution (extended to $R^{2}$; see *Materials and Methods*). For comparison, we also fitted a bivariate *t*-distribution (with the same number of parameters), as has been used to model natural image statistics (59, 88–90). Fig. 3*A* shows the distribution of the resulting log-likelihoods. The fact that the log-likelihoods of the Pareto model are greater than those of the multivariate *t* model (means: $-5.19\times 10^{6}$ for Pareto and $-5.65\times 10^{6}$ for *t*-distribution) suggests that the Pareto distribution is a strong contender as a model of natural image statistics. However, we do not consider our analysis to be definitive, and further research is required to determine how well the Pareto distribution describes image statistics quantitatively and qualitatively. But this analysis does demonstrate that the Pareto distribution, which we derived from first principles, is also likely to be an empirically relevant description of natural stimulus statistics.

**Fig. 4.**
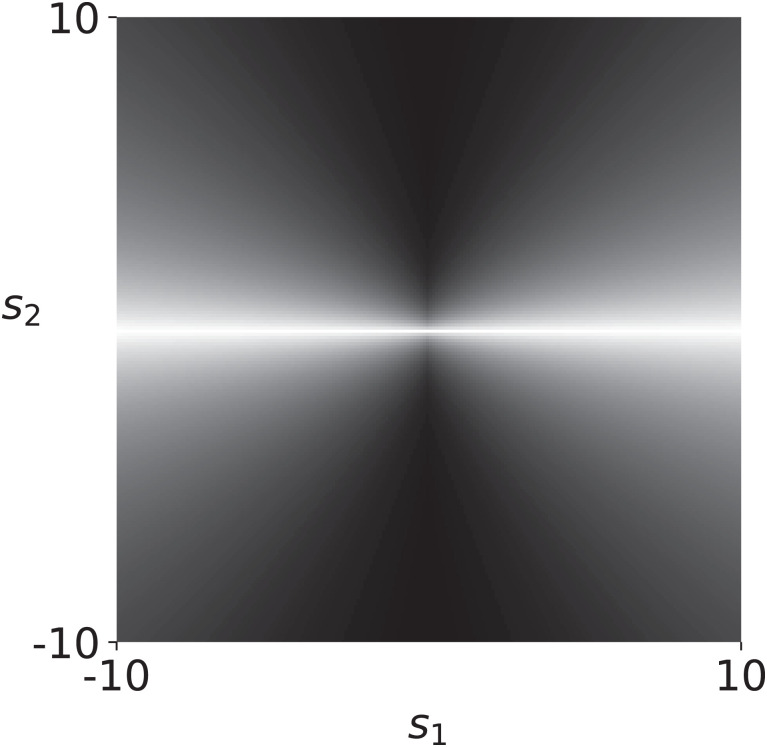
Conditional histogram of a bivariate Pareto distribution extended to $R^{2}$, with μ=0, σ=1, and *β* = 1. Brightness is proportional to the probability of *s*_2_ conditional on *s*_1_, rescaled in each column to use the full range of intensities, as is customary in the literature. The statistical dependence closely resembles the bow-tie shape observed empirically for many naturalistic stimuli (35).

Fig. 4 illustrates why the Pareto distribution may describe natural stimulus statistics well. The depicted conditional histogram of a bivariate Pareto type III distribution with *β* = 1 (extended to $R^{2}$) closely matches the empirically observed statistical dependence in filter responses to natural images. We conclude that the efficiently encoded Pareto distribution exhibits key features of naturalistic stimulus distributions, including this kind of statistical dependence as well as heavy-tailed marginal distributions. We further note that the average estimate for *β* was 1.2 (*SI Appendix*, Fig. S1), which is comparable to estimates of the divisive normalization exponent (*α*) from neural data (6). This may suggest that the divisive normalization parameter *α* is indeed tuned to the shape parameter *β* of the Pareto distribution.

### Relation to Existing Models of Natural Stimulus Statistics.

This section explores the connections to existing models of natural stimulus statistics, with the goal of providing a foundation for future research. We first show how the statistical dependence of the Pareto distribution can capture the conditional variance dependence that is commonly observed in bow-tie plots (86). To see this, assume that $S_{1},\ldots ,S_{n}$ are filter responses that are distributed according to a multivariate Pareto type III distribution. The variance of a filter response *S_i_* conditional on all other filter responses is then, for *β* = 1 (assumed for tractability) and *n* > 2, given by

[12] $$\begin{array}{l} \text{Var}(S_{i}|{\left\{S_{j}=s_{j}\right\}}_{j\neq i})\\ \;=\frac{\sigma_{i}^{2}n}{{\left(n-1\right)}^{2}(n-2)}{\left[1+\sum_{j\neq i}\left(\frac{s_{j}-\mu_{j}}{\sigma_{j}}\right)\right]}^{2}\\ \;=\frac{\sigma_{i}^{2}n}{{\left(n-1\right)}^{2}(n-2)}[1+2\sum_{j\neq i}\frac{s_{j}-\mu_{j}}{\sigma_{j}}+\\ \;\;\sum_{j\neq i}\sum_{k\neq i,k\neq j}\frac{s_{j}-\mu_{j}}{\sigma_{j}}\frac{s_{k}-\mu_{k}}{\sigma_{k}}+\sum_{j\neq i}{\left(\frac{s_{j}-\mu_{j}}{\sigma_{j}}\right)}^{2}]. \end{array}$$

The conditional variance dependence is thus quadratic, in accordance with Schwartz and Simoncelli (35) who use a similar quadratic model of the variance dependence observed in conditional histograms. Unlike in their model, the conditional distribution giving rise to this variance dependence is not Gaussian. The conditional distribution of the Pareto distribution in Eq. **6** has cdf

[13] $$\begin{array}{ll} F_{S_{i}|{\left\{S_{j}=s_{j}\right\}}_{j\neq i}}(s_{i};{\left\{s_{j}\right\}}_{j\neq i}, & \mu ,\sigma ,\beta )=\\ \; & 1-{\left[1+\frac{{\left(\frac{s_{i}-\mu_{i}}{\sigma_{i}}\right)}^{\beta }}{1+\sum_{j\neq i}{\left(\frac{s_{j}-\mu_{j}}{\sigma_{j}}\right)}^{\beta }}\right]}^{-n} \end{array}$$

for $s_{i}>\mu_{i}$, as we show in *SI Appendix*.^§^ For suitable parameter values this reduces to a log-logistic distribution, closely related to the conditionally lognormal distribution used by Wainwright et al. (55).

Next, we show that the Pareto distribution also has a connection with models of image statistics based on Gamma-weighted scale mixtures of Gaussian random variables (60). It turns out that the Pareto type III distribution can be expressed as a Gamma mixture of transformed exponential (or Weibull) random variables (ref. 80, chap. 6.2). The following proposition shows that the efficiently coded distribution of Eq. **6** is a particularly simple mixture of transformed standard exponential random variables:

Proposition 4. *Let* $U_{i}\overset{\mathrm{iid}}{\sim }\text{Exp}(\lambda =1)$ *for* i=1,…,n, *and let* Z~Exp(λ=1) *independently of all U_i_. Then the distribution of* $S=(S_{1},\ldots ,S_{n})$ *with*

[14] $$S_{i}=\mu_{i}+\sigma_{i}{\left(U_{i}/Z\right)}^{1/\beta }\;\;\text{for}\;i=1,\ldots ,n,$$

*is a Pareto type III distribution with the joint survival function* ${\bar{F}}_{S}(s_{1},\ldots ,s_{n};\mu ,\sigma ,\beta )$ *of* *Eq*. **6**.

This result not only facilitates comparisons with existing models of naturalistic stimuli, it is also of practical importance, since it provides a simple way to draw samples from the multivariate Pareto type III distribution.

## Discussion

We have characterized the family of stimulus distributions for which divisive normalization maximizes mutual information in the low-noise limit. Absent metabolic costs, this family consists of particular multivariate Pareto type III distributions, which divisive normalization transforms into an entropy-maximizing output distribution. Taking into account metabolic costs of an arbitrary shape, the efficiently encoded input distributions take a generalized form in which inputs resulting in costlier outputs occur less frequently. We note that our result does not imply that divisive normalization is the only neural encoding to satisfy the necessary and sufficient condition for efficiency, even with a Pareto stimulus distribution. Rather, any encoding whose Jacobian satisfies, given some input distribution, the equivalent of Eq. **4** in *Theorem 1* attains the same output distribution and will thus be equally efficient. Yet for divisive normalization to be efficient, the stimulus distribution must necessarily be of the prescribed type.

We further demonstrated that the Pareto distribution is consistent with empirical findings on naturalistic stimulus statistics. In the context of vision, the Pareto distribution captures key features observed in the statistics of natural images that are commonly modeled with Gaussian scale mixtures (60, 91). Our empirical analysis demonstrated that the Pareto distribution may be a similarly good model of natural-image statistics. *Proposition 4* identifies the Pareto distribution as a mixture of transformed exponential random variables and may thus prove helpful in further exploring relations to existing Gaussian mixture models of natural stimuli. While sharing some features with such models, the Pareto distribution differs in other respects. For example, unlike Gaussian scale mixtures, it is not elliptically symmetric. This means that it can be skewed but is not invariant to rotations of the coordinate system and may thus capture joint histograms resembling a diamond or rhombus rather than an ellipse. Furthermore, the Pareto distribution also captures features of typical luminance and contrast distributions (92). We leave exploring these issues for future research.

*Proposition 4* also opens the door to future work exploring more general coding mechanisms that are efficient for a wider class of input distributions. The distribution we find to be efficiently encoded is a special case of a Pareto type III distribution that is restrictive in the sense that a single shape parameter *β* governs the distribution’s dependence structure as well as the shape of its marginals. *Proposition 4* suggests that another fruitful direction, beyond considering more general Pareto distributions, may be to consider mixtures of an exponential random vector whose components are not independent, allowing for a more general covariance structure.

It would also be desirable to generalize our result to allow normalization weights to differ across pairs of neurons. Ruling out the case where different neurons in a population interact with different weights eliminates the possibility that neurons representing more proximal inputs normalize each other more strongly than those representing more distant inputs. Such distance-dependent normalization would be an efficient code of stimuli whose statistics reflect a notion of proximity. For example, this is the case for contrast in images, where correlation is decreasing as a function of distance. Our current framework does not incorporate such a distance notion for reasons of analytical tractability, but an extension would be of interest.

Our results provide a sharp testable prediction that can potentially be used to test empirically the efficient coding hypothesis. In a neural system whose response function is well described by divisive normalization, testing whether the inputs are Pareto distributed amounts to testing a necessary condition for the efficient coding hypothesis to hold. A more rigorous and demanding test would also estimate the normalization parameters as well as the parameters of the distribution and test whether they satisfy the conditions imposed by *Theorems 2* and *3*. Formally, if the efficient coding hypothesis holds, then the hypothesis that such a system’s inputs are Pareto distributed with α=β and $\lambda_{i}={\left(b/\sigma_{i}\right)}^{\alpha }$ must be true (under the maintained hypothesis of constant metabolic costs). Rejecting this null hypothesis would thus result in the rejection of the efficient coding hypothesis. Experiments that systematically manipulate the distribution of sensory stimuli (or choice sets) in a subject’s environment could even examine whether the divisive normalization parameters (such as *α*) optimally adapt to different stimulus distribution contexts (e.g., Pareto distributions with different values for *β*). Of course, less demanding tests are possible and informative.

It is important to stress that according to *Theorem 2*, testing the input distribution is a test of the joint hypothesis that coding is efficient and metabolic costs are of a particular form. For instance, Pareto-distributed inputs are only necessary for the efficient coding hypothesis under the maintained assumption of constant metabolic costs. This is a feature, not a bug. *Theorem 2* can be viewed as a representation theorem, analogous to the economics tradition of testing whether choice behavior is consistent with maximizing some utility function. This provides additional degrees of freedom: If the input distribution adheres to Eq. **5** for some metabolic cost function, then the efficient coding hypothesis would not be rejected.

Our work raises the possibility that divisive normalization is ubiquitous because it is an adaptation to Pareto distributions that are themselves widespread—in stimulus statistics but also, perhaps, in the statistics of other quantities. One example of such a quantity is firing rates of neurons, which in many neural systems appear to follow lognormal distributions (93–95), whose heavy-tailed shape closely resembles that of Pareto distributions. If firing rates of an upstream system are approximately Pareto distributed, it is conceivable that divisive normalization may be an adaptation to the firing-rate distribution of neurons from which it receives input. But, of course, the resulting outputs would then not be Pareto distributed, raising the question of whether the observed cascade of normalization-like modules in the brain is to some extent redundant. If each stage in a series of divisive normalization levels in the brain receives the preceding stage’s output as its input, then each stage faces a different distributional structure. Understanding how several stages of divisive normalization may work together to produce an efficient code is an open issue that is beyond the scope of this paper. Perhaps, applying our result to distributions of firing rates will help shed light on such issues.

The fact that divisive normalization has been implicated in settings beyond sensory processing and perception, particularly in value representations (17, 18) and choice (19, 21), raises the question of whether the Pareto distribution is also an ecologically relevant description of value-based choice environments. The univariate Pareto distribution of Eq. **8** (with *μ* = 0) is often used to model wealth and income distributions in economics, which is where it originated (96, 97). Moreover, the Pareto distribution is intimately related to Zipf’s law and is observed in many complex systems (82). Interestingly, it is also remarkably consistent (98) with Benford’s law (99, 100), according to which the leading digits in many naturally arising sets of numbers are likely to be small.

The heavy-tailed power-law characteristic of the Pareto distribution is relevant not only in sensory contexts, but also in many economic contexts (101, 102) ranging from city-size distributions to stock returns and trading volumes (103). While empirical evidence from choice environments is lacking, the widespread occurrence of Pareto distributions in economic contexts hints that divisive normalization might be observed in value representations and choice behavior because it is an efficient code of Pareto-distributed values within choice sets. An important caveat to this hypothesis is that mutual information is less relevant as an objective function for a decision maker than it is in the sensory domain. The efficient code for a utility-maximizing decision maker differs from the information-maximizing code (104–106). Conditions under which divisive normalization is efficient for choice remain to be determined.

In conclusion, our theoretical results make a simple and sharp prediction about why divisive normalization may be observed across a wide range of settings. This prediction can be tested in different domains, ranging from electrophysiological studies to empirical studies of value distributions and experiments on choice behavior. Our findings provide a framework for future research to test empirically and experimentally whether divisive normalization occurs in environments in which it is an efficient computation.

## Materials and Methods

All proofs are given in *SI Appendix*. The empirical analysis examined the statistics of filter responses to naturalistic images. For a set of 100 images from the (linear) van Hateren image dataset (87), we computed filter responses using a steerable pyramid (107) with four levels and four bands differing in orientation, using the matlabPyrTools package. (See https://github.com/LabForComputationalVision/matlabPyrTools and www.cns.nyu.edu/~eero/STEERPYR/ for more information.) We examined the statistical dependency between a pair of filters of the pyramid’s second level with orientations of 45^∘^ and 135^∘^ counterclockwise, respectively (108, figure 1.9). For each image separately, we fitted the joint distribution of these filter responses by obtaining maximum-likelihood estimates of the parameters of two bivariate distributions. The first distribution is a Pareto type III distribution extended to $R^{2}$, in the sense that the density at any $s\in R^{2}$ is given by $\frac{1}{4}f_{S}(|s|;\mu =0,\sigma ,\beta )$, where $f_{S}$ is as in Eq. **7**. We imposed the restriction $\sigma_{1}=\sigma_{2}$ to ensure that the number of free parameters is not larger than for the second distribution, which was a bivariate *t*-distribution with correlation parameter *c* and degrees of freedom *df*. The estimation was performed using the mle function of Matlab R2022a.

## Supplementary Material

Supplementary File

## Data Availability

Computer code can be accessed on GitHub (https://github.com/stefan-f-bucher/divisive-normalization-efficiency) (109). The van Hateren image dataset (87) is available at https://pirsquared.org/research/vhatdb/.

## Footnotes

^*^Our method extends to other formalizations of the efficient coding principle that relax the small-noise assumption (65), since we provide an analytical formula relating any input distribution to the distribution of its representation.

^†^Other, more general approximations are possible (65) but, for mathematical simplicity, are not considered here. However, *Theorem 1* is general and could be combined with variants of *Proposition 2*.

^‡^The minus sign in ref. 80, equation 6.1.17 appears to be a typo.

^§^Note that the conditional distribution is a univariate Pareto distribution of type IV (rather than III). Pareto type III distributions are not closed under conditioning.
