# Supplementary material for: Divisive normalization is an efficient code for multivariate Pareto-distributed environments
Source: Proc Natl Acad Sci U S A. 2022 Sep 26;119(40):e2120581119. doi: 10.1073/pnas.2120581119 (PMC9546555; doi:10.1073/pnas.2120581119)
Supplement: Supplementary File [file pnas.2120581119.sapp.pdf]

PNAS
www.pnas.org

Supplementary Information for

Divisive normalization is an efficient code for multivariate Pareto-distributed environments

Stefan Bucher and Adam Brandenburger

Corresponding author: Stefan Bucher.
E-mail: stefan.bucher@nyu.edu

This PDF file includes:

Supplementary text
Figs. S1 to S9
SI References

Supporting Information Text

Equation numbers without an "A" prefix refer to the main text.

Proof of Proposition 1. To see that $\boldsymbol{r}(\boldsymbol{x}) \in \Delta$ for all $\boldsymbol{x} \in \mathbb{R}_+^n$ (non-negative real vectors), note that

$$\sum_{i=1}^{n} \lambda_i r_i(\boldsymbol{x}) = \sum_{i=1}^{n} \lambda_i \gamma \frac{x_i^\alpha}{b^\alpha + \sum_{j=1}^n \lambda_j x_j^\alpha} = \gamma \frac{\sum_{i=1}^n \lambda_i x_i^\alpha}{b^\alpha + \sum_{j=1}^n \lambda_j x_j^\alpha} < \gamma.$$

We next show that for all $\boldsymbol{y} \in \Delta$ there is a unique $\boldsymbol{x} \in \mathbb{R}_+^n$ such that $\boldsymbol{r}(\boldsymbol{x}) = \boldsymbol{y}$, i.e., such that

$$\gamma \frac{x_i^\alpha}{b^\alpha + \sum_{j=1}^n \lambda_j x_j^\alpha} = y_i,$$

for all i. Letting $z_i = \lambda_i x_i^\alpha$, $w_i = \lambda_i y_i / \gamma$, and $\tilde{b} = b^\alpha$, this is equivalent to showing that there is a unique $\boldsymbol{z} \in \mathbb{R}_+^n$ such that

$$\frac{z_i}{\tilde{b} + \sum_{j=1}^n z_j} = w_i,$$

for all i, or, equivalently, that the system of equations

$$\left(\mathbf{I}_n - \boldsymbol{w}\mathbf{1}^T\right) \boldsymbol{z} = \tilde{b}\boldsymbol{w} \qquad \text{[A.1]}$$

has a unique solution $\boldsymbol{z} \in \mathbb{R}_+^n$. The matrix determinant lemma (1, Theorem 18.1.1) states that for an invertible square matrix $\boldsymbol{A}$ and column vectors $\boldsymbol{u}$ and $\boldsymbol{v}$,

$$\det(\boldsymbol{A} + \boldsymbol{u}\boldsymbol{v}^T) = (1 + \boldsymbol{v}^T \boldsymbol{A}^{-1} \boldsymbol{u}) \det(\boldsymbol{A}). \qquad \text{[A.2]}$$

Setting $\boldsymbol{A} = \mathbf{I}_n$, $\boldsymbol{u} = -\boldsymbol{w}$, and $\boldsymbol{v} = \mathbf{1}$, we then get

$$\det(\mathbf{I}_n - \boldsymbol{w}\mathbf{1}^T) = (1 - \mathbf{1}^T \mathbf{I}_n^{-1} \boldsymbol{w}) \det(\mathbf{I}_n) = 1 - \sum_{i=1}^n w_i > 0,$$

since $\sum_{i=1}^n w_i = \frac{1}{\gamma} \sum_{i=1}^n \lambda_i y_i < 1$ for all $\boldsymbol{y} \in \Delta$. Therefore, the system of equations A.1 indeed has a unique solution, so $\boldsymbol{r}$ is invertible and its image is Δ.

Proof of Proposition 2. First note that (2, Eq. 8.48)

$$I(\boldsymbol{X}; \boldsymbol{g}(\boldsymbol{X}) + \boldsymbol{\varepsilon}) = h(\boldsymbol{g}(\boldsymbol{X}) + \boldsymbol{\varepsilon}) - h(\boldsymbol{g}(\boldsymbol{X}) + \boldsymbol{\varepsilon} | \boldsymbol{X}) = h(\boldsymbol{g}(\boldsymbol{X}) + \boldsymbol{\varepsilon}) - h(\boldsymbol{\varepsilon} | \boldsymbol{X}). \qquad \text{[A.3]}$$

It follows that maximizing $I(\boldsymbol{X}; \boldsymbol{g}(\boldsymbol{X}) + \boldsymbol{\varepsilon})$ is equivalent to maximizing $h(\boldsymbol{g}(\boldsymbol{X}) + \boldsymbol{\varepsilon})$ (cf. 3, 4).

For small noise, maximizing $I(\boldsymbol{X}; \boldsymbol{g}(\boldsymbol{X}) + \boldsymbol{\varepsilon})$ is approximately equivalent to maximizing $h(\boldsymbol{g}(\boldsymbol{X}))$. To see that this is an arbitrarily close approximation, note that by repeated application of the chain rule (2, Theorem 17.2.2 and Problem 2.4)*

$$\begin{aligned} h(\boldsymbol{g}(\boldsymbol{X}) + \boldsymbol{\varepsilon}) &\leq h(\boldsymbol{g}(\boldsymbol{X}) + \boldsymbol{\varepsilon}) + h(\boldsymbol{g}(\boldsymbol{X}), \boldsymbol{\varepsilon} | \boldsymbol{g}(\boldsymbol{X}) + \boldsymbol{\varepsilon}) \\ &= h(\boldsymbol{g}(\boldsymbol{X}), \boldsymbol{\varepsilon}, \boldsymbol{g}(\boldsymbol{X}) + \boldsymbol{\varepsilon}) \\ &= h(\boldsymbol{g}(\boldsymbol{X}), \boldsymbol{\varepsilon}) + h(\boldsymbol{g}(\boldsymbol{X}) + \boldsymbol{\varepsilon} | \boldsymbol{g}(\boldsymbol{X}), \boldsymbol{\varepsilon}) \\ &= h(\boldsymbol{g}(\boldsymbol{X}), \boldsymbol{\varepsilon}) \\ &= h(\boldsymbol{g}(\boldsymbol{X})) + h(\boldsymbol{\varepsilon}), \end{aligned}$$

where the last line uses independence of $\boldsymbol{g}(\boldsymbol{X})$ and $\boldsymbol{\varepsilon}$, which further implies (2, Lemma 17.2.1)

$$h(\boldsymbol{g}(\boldsymbol{X}) + \boldsymbol{\varepsilon}) \geq h(\boldsymbol{g}(\boldsymbol{X})),$$

so that

$$h(\boldsymbol{g}(\boldsymbol{X})) \leq h(\boldsymbol{g}(\boldsymbol{X}) + \boldsymbol{\varepsilon}) \leq h(\boldsymbol{g}(\boldsymbol{X})) + h(\boldsymbol{\varepsilon}).$$

By independence we have $h(\boldsymbol{\varepsilon} | \boldsymbol{X}) = h(\boldsymbol{\varepsilon})$ (2, Theorem 17.2.1), so it follows from Equation A.3 that

$$I(\boldsymbol{X}; \boldsymbol{g}(\boldsymbol{X}) + \boldsymbol{\varepsilon}) = h(\boldsymbol{g}(\boldsymbol{X}) + \boldsymbol{\varepsilon}) - h(\boldsymbol{\varepsilon}),$$

so that

$$h(\boldsymbol{g}(\boldsymbol{X})) - h(\boldsymbol{\varepsilon}) \leq I(\boldsymbol{X}; \boldsymbol{g}(\boldsymbol{X}) + \boldsymbol{\varepsilon}) \leq h(\boldsymbol{g}(\boldsymbol{X})),$$

and thus

$$|I(\boldsymbol{X}; \boldsymbol{g}(\boldsymbol{X}) + \boldsymbol{\varepsilon}) - h(\boldsymbol{g}(\boldsymbol{X}))| \leq h(\boldsymbol{\varepsilon}) < \delta,$$

for any $\delta > 0$, as long as $h(\boldsymbol{\varepsilon}) < \delta$.

*This generalizes to any function $\boldsymbol{f}(\boldsymbol{g}(\boldsymbol{X}), \boldsymbol{\varepsilon})$ beyond $\boldsymbol{f}(\boldsymbol{g}(\boldsymbol{X}), \boldsymbol{\varepsilon}) = \boldsymbol{g}(\boldsymbol{X}) + \boldsymbol{\varepsilon}$.

Proof of Proposition 3. We want to show that the pdf $f_{\boldsymbol{Y}}$ of Equation 3, which equivalently satisfies

$$\log f_{\boldsymbol{Y}}(\boldsymbol{y}) = \lambda_0 - c(\boldsymbol{y}) \qquad \forall \boldsymbol{y} \in \mathcal{C},$$

with $\lambda_0 = -\log \int_{\mathcal{C}} e^{-c(\boldsymbol{z})} d\boldsymbol{z}$, uniquely maximizes

$$h(\boldsymbol{Y}) - \mathbb{E}[c(\boldsymbol{Y})] = -\int_{\mathcal{C}} g(\boldsymbol{y}) \log g(\boldsymbol{y}) d\boldsymbol{y} - \int_{\mathcal{C}} g(\boldsymbol{y}) c(\boldsymbol{y}) d\boldsymbol{y}$$

among all pdfs g satisfying

$$\begin{aligned} g(\boldsymbol{y}) &\geq 0 \quad \text{with equality for all } \boldsymbol{y} \notin \mathcal{C}, \\ \int_{\mathcal{C}} g(\boldsymbol{y}) d\boldsymbol{y} &= 1. \end{aligned}$$

Our proof parallels Theorem 12.1.1. of Cover and Thomas (2). Recall that the Kullback-Leibler divergence satisfies (2, eq. 8.87)

$$D\left(g || f_{\boldsymbol{Y}}\right) = \int_{\mathcal{C}} g(\boldsymbol{y}) \log \left(\frac{g(\boldsymbol{y})}{f_{\boldsymbol{Y}}(\boldsymbol{y})} \right) d\boldsymbol{y} \geq 0,$$

and note that

$$\begin{aligned} -\int_{\mathcal{C}} g(\boldsymbol{y}) \log g(\boldsymbol{y}) d\boldsymbol{y} - \int_{\mathcal{C}} g(\boldsymbol{y}) c(\boldsymbol{y}) d\boldsymbol{y} &= -\int_{\mathcal{C}} g(\boldsymbol{y}) \log \left(\frac{g(\boldsymbol{y})}{f_{\boldsymbol{Y}}(\boldsymbol{y})} f_{\boldsymbol{Y}}(\boldsymbol{y}) \right) d\boldsymbol{y} - \int_{\mathcal{C}} g(\boldsymbol{y}) c(\boldsymbol{y}) d\boldsymbol{y} \\ &= -\int_{\mathcal{C}} g(\boldsymbol{y}) \log \left(\frac{g(\boldsymbol{y})}{f_{\boldsymbol{Y}}(\boldsymbol{y})} \right) d\boldsymbol{y} - \int_{\mathcal{C}} g(\boldsymbol{y}) \log \left(f_{\boldsymbol{Y}}(\boldsymbol{y}) \right) d\boldsymbol{y} - \int_{\mathcal{C}} g(\boldsymbol{y}) c(\boldsymbol{y}) d\boldsymbol{y} \\ &= -D\left(g || f_{\boldsymbol{Y}}\right) - \int_{\mathcal{C}} g(\boldsymbol{y}) \log \left(f_{\boldsymbol{Y}}(\boldsymbol{y}) \right) d\boldsymbol{y} - \int_{\mathcal{C}} g(\boldsymbol{y}) c(\boldsymbol{y}) d\boldsymbol{y} \\ &\leq -\int_{\mathcal{C}} g(\boldsymbol{y}) \left[\log \left(f_{\boldsymbol{Y}}(\boldsymbol{y}) \right) + c(\boldsymbol{y}) \right] d\boldsymbol{y} \\ &= -\lambda_0 \int_{\mathcal{C}} g(\boldsymbol{y}) d\boldsymbol{y} \\ &= -\lambda_0 \int_{\mathcal{C}} f_{\boldsymbol{Y}}(\boldsymbol{y}) d\boldsymbol{y} \\ &= -\int_{\mathcal{C}} f_{\boldsymbol{Y}}(\boldsymbol{y}) \left[\lambda_0 - c(\boldsymbol{y}) + c(\boldsymbol{y}) \right] d\boldsymbol{y} \\ &= -\int_{\mathcal{C}} f_{\boldsymbol{Y}}(\boldsymbol{y}) \log \left(f_{\boldsymbol{Y}}(\boldsymbol{y}) \right) d\boldsymbol{y} - \int_{\mathcal{C}} f_{\boldsymbol{Y}}(\boldsymbol{y}) c(\boldsymbol{y}) d\boldsymbol{y}, \end{aligned}$$

where the inequality is strict unless $f_{\boldsymbol{Y}} = g$ almost everywhere (2, Theorem 8.6.1). We have shown that the pdf $f_{\boldsymbol{Y}}$ of Equation 3 attains strictly greater $h(\boldsymbol{Y}) - \mathbb{E}[c(\boldsymbol{Y})]$ than any other pdf with support $\mathcal{C}$.

Proof of Theorem 1. Since the function $\boldsymbol{r}$ is invertible and has continuous derivatives, a change of variables implies that

$$f_{\boldsymbol{X}}(\boldsymbol{x}) = f_{\boldsymbol{Y}}(\boldsymbol{r}(\boldsymbol{x})) \cdot \left| \det \left(\boldsymbol{J}_{\boldsymbol{r}}(\boldsymbol{x}) \right) \right| \qquad \forall \boldsymbol{x} \in \mathbb{R}_+^n,$$

whenever the determinant of the Jacobian $\boldsymbol{J}_{\boldsymbol{r}}(\boldsymbol{x})$ of $\boldsymbol{r}$ is non-zero (5, Theorem 8.1.7).[†] We first compute the Jacobian of $\tilde{\boldsymbol{r}}(\tilde{\boldsymbol{x}}) = \gamma \tilde{\boldsymbol{x}} / d(\tilde{\boldsymbol{x}})$ where $d(\tilde{\boldsymbol{x}}) = b^\alpha + \boldsymbol{\lambda}^T \tilde{\boldsymbol{x}}$:

$$\boldsymbol{J}_{\tilde{\boldsymbol{r}}}(\tilde{\boldsymbol{x}}) = \frac{\gamma}{d(\tilde{\boldsymbol{x}})^2} \cdot \begin{pmatrix} d(\tilde{\boldsymbol{x}}) - \tilde{x}_1 \lambda_1 & -\tilde{x}_1 \lambda_2 & \cdots & -\tilde{x}_1 \lambda_n \\ -\tilde{x}_2 \lambda_1 & d(\tilde{\boldsymbol{x}}) - \tilde{x}_2 \lambda_2 & \cdots & -\tilde{x}_2 \lambda_n \\ \vdots & \vdots & \ddots & \vdots \\ -\tilde{x}_n \lambda_1 & -\tilde{x}_n \lambda_2 & \cdots & d(\tilde{\boldsymbol{x}}) - \tilde{x}_n \lambda_n \end{pmatrix} = \frac{\gamma}{d(\tilde{\boldsymbol{x}})^2} \cdot \left(d(\tilde{\boldsymbol{x}}) \cdot \mathbf{I}_n - \tilde{\boldsymbol{x}} \boldsymbol{\lambda}^T \right),$$

[†] The relation extends to include the boundary of the domain $\mathbb{R}_+^n$ (6, Corollary 15.9) and could alternatively be stated on $\mathbb{R}^n$.

where $\mathbf{I}_n$ is the $n \times n$ identity matrix. Using the matrix determinant lemma (Eq. A.2), with $\boldsymbol{A} = d(\tilde{\boldsymbol{x}}) \cdot \mathbf{I}_n$, $\boldsymbol{u} = -\tilde{\boldsymbol{x}}$, and $\boldsymbol{v} = \boldsymbol{\lambda}$, we obtain

$$
\begin{aligned}
\det\left(\boldsymbol{J}_{\tilde{\boldsymbol{r}}}\left(\tilde{\boldsymbol{x}}\right)\right) &= \left(\frac{\gamma}{d(\tilde{\boldsymbol{x}})^2}\right)^n \det\left(d(\tilde{\boldsymbol{x}}) \cdot \mathbf{I}_n - \tilde{\boldsymbol{x}}\boldsymbol{\lambda}^T\right) \\
&= \left(\frac{\gamma}{d(\tilde{\boldsymbol{x}})^2}\right)^n \left(1 - \boldsymbol{\lambda}^T \left(d(\tilde{\boldsymbol{x}}) \cdot \mathbf{I}_n\right)^{-1} \tilde{\boldsymbol{x}}\right) \det\left(d(\tilde{\boldsymbol{x}}) \cdot \mathbf{I}_n\right) \\
&= \left(\frac{\gamma}{d(\tilde{\boldsymbol{x}})^2}\right)^n \left(1 - \frac{\boldsymbol{\lambda}^T\tilde{\boldsymbol{x}}}{d(\tilde{\boldsymbol{x}})}\right) d(\tilde{\boldsymbol{x}})^n \\
&= \left(\frac{\gamma}{d(\tilde{\boldsymbol{x}})^2}\right)^n \left(1 - \frac{\boldsymbol{\lambda}^T\tilde{\boldsymbol{x}}}{b^\alpha + \boldsymbol{\lambda}^T\tilde{\boldsymbol{x}}}\right) d(\tilde{\boldsymbol{x}})^n \\
&= \left(\frac{\gamma}{d(\tilde{\boldsymbol{x}})^2}\right)^n \left(\frac{b^\alpha}{d(\tilde{\boldsymbol{x}})}\right) d(\tilde{\boldsymbol{x}})^n \\
&= \gamma^n \frac{b^\alpha}{\left(b^\alpha + \boldsymbol{\lambda}^T\tilde{\boldsymbol{x}}\right)^{n+1}}.
\end{aligned}
$$

In order to find the Jacobian of $\boldsymbol{r}(\boldsymbol{x}) = \tilde{\boldsymbol{r}}(\boldsymbol{x}^\alpha)$, note that by the multivariate chain rule:

$$
\left(\mathbf{J}_{\boldsymbol{r}}(\boldsymbol{x})\right)_{ij} = \frac{\partial}{\partial x_j}\tilde{\boldsymbol{r}}_i\left(\boldsymbol{x}^\alpha\right) = \sum_k \frac{\partial}{\partial \tilde{x}_k}\tilde{\boldsymbol{r}}_i\left(\tilde{\boldsymbol{x}}\right)\frac{\partial}{\partial x_j}x_k^\alpha = \frac{\partial \tilde{\boldsymbol{r}}_i\left(\tilde{\boldsymbol{x}}\right)}{\partial \tilde{x}_j}\alpha x_j^{\alpha-1} = \left(\boldsymbol{J}_{\tilde{\boldsymbol{r}}}(\tilde{\boldsymbol{x}})\right)_{ij}\alpha x_j^{\alpha-1},
$$

so that

$$
\mathbf{J}_{\boldsymbol{r}}(\boldsymbol{x}) = \frac{\gamma}{d(\boldsymbol{x}^\alpha)^2} \cdot \begin{pmatrix}
(d(\boldsymbol{x}^\alpha) - x_1^\alpha\lambda_1)\cdot\alpha x_1^{\alpha-1} & (-x_1^\alpha\lambda_2)\cdot\alpha x_2^{\alpha-1} & \cdots & (-x_1^\alpha\lambda_n)\cdot\alpha x_n^{\alpha-1} \\
(-x_2^\alpha\lambda_1)\cdot\alpha x_1^{\alpha-1} & (d(\boldsymbol{x}^\alpha) - x_2^\alpha\lambda_2)\cdot\alpha x_2^{\alpha-1} & \cdots & (-x_2^\alpha\lambda_n)\cdot\alpha x_n^{\alpha-1} \\
\vdots & \vdots & \ddots & \vdots \\
(-x_n^\alpha\lambda_1)\cdot\alpha x_1^{\alpha-1} & (-x_n^\alpha\lambda_2)\cdot\alpha x_2^{\alpha-1} & \cdots & (d(\boldsymbol{x}^\alpha) - x_n^\alpha\lambda_n)\cdot\alpha x_n^{\alpha-1}
\end{pmatrix}
$$

and, therefore,

$$
\det\left(\mathbf{J}_{\boldsymbol{r}}(\boldsymbol{x})\right) = \det\left(\boldsymbol{J}_{\tilde{\boldsymbol{r}}}\left(\boldsymbol{x}^\alpha\right)\right)\cdot\alpha^n\prod_{i=1}^n x_i^{\alpha-1} = \gamma^n\alpha^n\frac{b^\alpha\prod_{i=1}^n x_i^{\alpha-1}}{\left(b^\alpha + \sum_{i=1}^n \lambda_i x_i^\alpha\right)^{n+1}}.
$$

Since $\det\left(\mathbf{J}_{\boldsymbol{r}}(\boldsymbol{x})\right) > 0$ for all $\boldsymbol{x} \in \mathbb{R}^n_{++}$ we then have

$$
f_{\boldsymbol{X}}(\boldsymbol{x}) = f_{\boldsymbol{Y}}(\boldsymbol{y})\cdot\gamma^n\alpha^n\frac{b^\alpha\prod_{i=1}^n x_i^{\alpha-1}}{\left(b^\alpha + \sum_{i=1}^n \lambda_i x_i^\alpha\right)^{n+1}}, \tag{4}
$$

for any positive vector $\boldsymbol{x} \in \mathbb{R}^n_{++}$.

Proof of Theorem 2. Note that the support of $\boldsymbol{r}(\boldsymbol{x})$ is its image which, by Proposition 1, is given by Δ. From Theorem 1 combined with Proposition 3 it follows that

$$
f_{\boldsymbol{X}}(\boldsymbol{x}) = \gamma^n\alpha^n\frac{b^\alpha\prod_{i=1}^n x_i^{\alpha-1}}{\left(b^\alpha + \sum_{i=1}^n \lambda_i x_i^\alpha\right)^{n+1}}\frac{\exp\left(-c\left(\boldsymbol{r}(\boldsymbol{x})\right)\right)}{\int_\Delta \exp\left(-c\left(\boldsymbol{z}\right)\right)d\boldsymbol{z}} \quad \forall \boldsymbol{x} \in \mathbb{R}^n_{++},
$$

is a necessary and sufficient condition for $\boldsymbol{r}$ to maximize the entropy of the output distribution net of expected costs over all representations with support Δ. Setting $\alpha \equiv \beta$ and $b/\lambda_i^{1/\alpha} \equiv \sigma_i$ we get

$$
\begin{aligned}
f_{\boldsymbol{X}}(\boldsymbol{x}) &= \gamma^n\beta^n\frac{b^\beta\prod_{i=1}^n x_i^{\beta-1}}{b^{\beta(n+1)}\left(1 + \sum_{i=1}^n (x_i/\sigma_i)^\beta\right)^{n+1}}\frac{\exp\left(-c\left(\boldsymbol{r}(\boldsymbol{x})\right)\right)}{\int_\Delta \exp\left(-c\left(\boldsymbol{z}\right)\right)d\boldsymbol{z}} \\
&= \gamma^n\beta^n\frac{\prod_{i=1}^n x_i^{\beta-1}/b^\beta}{\left(1 + \sum_{i=1}^n (x_i/\sigma_i)^\beta\right)^{n+1}}\frac{\exp\left(-c\left(\boldsymbol{r}(\boldsymbol{x})\right)\right)}{\int_\Delta \exp\left(-c\left(\boldsymbol{z}\right)\right)d\boldsymbol{z}} \quad \forall \boldsymbol{x} \in \mathbb{R}^n_{++}.
\end{aligned}
$$

The result follows from the fact that for any constant translation $\boldsymbol{\mu} \in \mathbb{R}^n$

$$
f_{\boldsymbol{S}}(\boldsymbol{s}) = f_{\boldsymbol{X}}(\boldsymbol{s} - \boldsymbol{\mu}) = \gamma^n\beta^n\frac{\prod_{i=1}^n \left(s_i - \mu_i\right)^{\beta-1}/b^\beta}{\left(1 + \sum_{i=1}^n\left(\frac{s_i-\mu_i}{\sigma_i}\right)^\beta\right)^{n+1}} \times \frac{\exp(-c(\boldsymbol{r}(\boldsymbol{s}-\boldsymbol{\mu})))}{\int_\Delta \exp(-c(\boldsymbol{z}))d\boldsymbol{z}} \qquad \forall \boldsymbol{s} > \boldsymbol{\mu}. \tag{5}
$$

Proof of Theorem 3. In the special case of constant costs $c(\boldsymbol{y}) = \bar{c}$ for all $\boldsymbol{y} \in \Delta$, Equation 5 of Theorem 2 reduces to

$$f_{\boldsymbol{S}}(\boldsymbol{s}) = \gamma^n \beta^n \frac{\prod_{i=1}^n (s_i - \mu_i)^{\beta-1}/b^\beta}{\left(1 + \sum_{i=1}^n \left(\frac{s_i-\mu_i}{\sigma_i}\right)^\beta\right)^{n+1}} \frac{\exp(-\bar{c})}{\int_\Delta \exp(-\bar{c})\, d\boldsymbol{z}} = \gamma^n \beta^n \frac{\prod_{i=1}^n \frac{1}{\lambda_i \sigma_i}\left(\frac{s_i-\mu_i}{\sigma_i}\right)^{\beta-1}}{\left(1 + \sum_{i=1}^n \left(\frac{s_i-\mu_i}{\sigma_i}\right)^\beta\right)^{n+1}} \frac{1}{\int_\Delta 1 d\boldsymbol{z}} \qquad \boldsymbol{s} > \boldsymbol{\mu},$$

where we have used $b^\beta = \lambda_i \sigma_i^\beta$ and where

$$\int_\Delta 1 d\boldsymbol{z} = \frac{\gamma^n}{\lambda_1 \lambda_2 \cdots \lambda_n \cdot n!},$$

so that

$$f_{\boldsymbol{S}}(\boldsymbol{s}) = \gamma^n \beta^n \frac{\prod_{i=1}^n \frac{1}{\lambda_i \sigma_i}\left(\frac{s_i-\mu_i}{\sigma_i}\right)^{\beta-1}}{\left(1 + \sum_{i=1}^n \left(\frac{s_i-\mu_i}{\sigma_i}\right)^\beta\right)^{n+1}} \frac{n!}{\gamma^n} \prod_{i=1}^n \lambda_i = \beta^n \frac{n! \prod_{i=1}^n \frac{1}{\sigma_i}\left(\frac{s_i-\mu_i}{\sigma_i}\right)^{\beta-1}}{\left(1 + \sum_{i=1}^n \left(\frac{s_i-\mu_i}{\sigma_i}\right)^\beta\right)^{n+1}} \qquad \boldsymbol{s} > \boldsymbol{\mu}. \qquad [7]$$

It remains to show that Equation 7 is the pdf of a multivariate Pareto type III distribution with joint survival function (7, Eq. 6.1.17 with $\gamma_i = 1/\beta$ for $i = 1, \ldots, n$),

$$\bar{F}_{\boldsymbol{S}}(\boldsymbol{s}; \boldsymbol{\mu}, \boldsymbol{\sigma}, \beta) = \left[1 + \sum_{i=1}^n \left(\frac{s_i - \mu_i}{\sigma_i}\right)^\beta\right]^{-1} \qquad \boldsymbol{s} > \boldsymbol{\mu}.^{\ddagger} \qquad [6]$$

To see this, define

$$\bar{F}_{\boldsymbol{S}}^{(k)}(s_1, \ldots, s_n; \boldsymbol{\mu}, \boldsymbol{\sigma}, \beta) \equiv \frac{\partial^k}{\partial s_1 \cdots \partial s_k} \bar{F}_{\boldsymbol{S}}(s_1, \ldots, s_n; \boldsymbol{\mu}, \boldsymbol{\sigma}, \beta) = \frac{\partial}{\partial s_k} \bar{F}_{\boldsymbol{S}}^{(k-1)}(s_1, \ldots, s_n; \boldsymbol{\mu}, \boldsymbol{\sigma}, \beta),$$

and note that, for $k = 0, 1, 2, \ldots, n$,

$$\bar{F}_{\boldsymbol{S}}^{(k)}(s_1, \ldots, s_n; \boldsymbol{\mu}, \boldsymbol{\sigma}, \beta) = (-1)^k \left(\prod_{i=1}^k \frac{\beta \cdot i}{\sigma_i} \left(\frac{s_i - \mu_i}{\sigma_i}\right)^{\beta-1}\right) \left[1 + \sum_{i=1}^n \left(\frac{s_i - \mu_i}{\sigma_i}\right)^\beta\right]^{-(k+1)}. \qquad [A.4]$$

This can be shown by induction. Note that $\bar{F}_{\boldsymbol{S}}^{(0)}(s_1, \ldots, s_n; \boldsymbol{\mu}, \boldsymbol{\sigma}, \beta) = \bar{F}_{\boldsymbol{S}}(s_1, \ldots, s_n; \boldsymbol{\mu}, \boldsymbol{\sigma}, \beta)$ so that Equation A.4 holds for $k = 0$, and observe that if Equation A.4 holds for $k-1$, then

$$\begin{aligned}
\bar{F}_{\boldsymbol{S}}^{(k)}(s_1, \ldots, s_n; \boldsymbol{\mu}, \boldsymbol{\sigma}, \beta) &= \frac{\partial}{\partial s_k} \bar{F}_{\boldsymbol{S}}^{(k-1)}(s_1, \ldots, s_n; \boldsymbol{\mu}, \boldsymbol{\sigma}, \beta) \\
&= \left((-1)^{k-1} \prod_{i=1}^{k-1} \frac{\beta \cdot i}{\sigma_i} \left(\frac{s_i - \mu_i}{\sigma_i}\right)^{\beta-1}\right) \frac{\partial}{\partial s_k} \left[1 + \sum_{i=1}^n \left(\frac{s_i - \mu_i}{\sigma_i}\right)^\beta\right]^{-k} \\
&= \left((-1)^{k-1} \prod_{i=1}^{k-1} \frac{\beta \cdot i}{\sigma_i} \left(\frac{s_i - \mu_i}{\sigma_i}\right)^{\beta-1}\right) \left(-k\beta \left(\frac{s_k - \mu_k}{\sigma_k}\right)^{\beta-1} \frac{1}{\sigma_k} \left[1 + \sum_{i=1}^n \left(\frac{s_i - \mu_i}{\sigma_i}\right)^\beta\right]^{-(k+1)}\right) \\
&= (-1)^k \left(\prod_{i=1}^k \frac{\beta \cdot i}{\sigma_i} \left(\frac{s_i - \mu_i}{\sigma_i}\right)^{\beta-1}\right) \left[1 + \sum_{i=1}^n \left(\frac{s_i - \mu_i}{\sigma_i}\right)^\beta\right]^{-(k+1)},
\end{aligned}$$

so that Equation A.4 also holds for k.

Next, note that, given $s_1, \ldots, s_n$ and denoting, for any i, the event $S_i > s_i$ by A_i and its complement by A_i^c, the cdf is obtained from the survival function as

$$\begin{aligned}
F_{\boldsymbol{S}}(s_1, \ldots, s_n) &= \mathbb{P}(S_1 \leq s_1, \ldots, S_n \leq s_n) \\
&= \mathbb{P}\left(\bigcap_{i=1}^n A_i^c\right) \\
&= 1 - \mathbb{P}\left(\bigcup_{i=1}^n A_i\right) \\
&= 1 - \sum_{k=1}^n (-1)^{k-1} \sum_{\substack{I \subseteq \{1,\ldots,n\} \\ |I|=k}} \mathbb{P}\left(\bigcap_{i \in I} A_i\right) \\
&= 1 + \sum_{k=1}^n (-1)^k \sum_{\substack{I \subseteq \{1,\ldots,n\} \\ |I|=k}} \lim_{s_i \to -\infty\, \forall i \notin I} \bar{F}_{\boldsymbol{S}}(s_1, \ldots, s_n),
\end{aligned}$$

‡The minus sign in (7, Eq. 6.1.17) appears to be a typo.

where the fourth equality follows from the probabilistic version of the inclusion-exclusion principle. Therefore

$$\begin{aligned}\frac{\partial^n}{\partial s_1\cdots\partial s_n}F_{\boldsymbol{S}}(s_1,\ldots,s_n) &= \frac{\partial^n}{\partial s_1\cdots\partial s_n}\left[1+\sum_{k=1}^{n}(-1)^k\sum_{\substack{I\subseteq\{1,\ldots,n\}\\|I|=k}}\lim_{s_i\to-\infty\,\forall i\notin I}\bar{F}_{\boldsymbol{S}}(s_1,\ldots,s_n)\right]\\ &= (-1)^n\frac{\partial^n}{\partial s_1\cdots\partial s_n}\bar{F}_{\boldsymbol{S}}(s_1,\ldots,s_n),\end{aligned}$$

so, using Equation A.4 with $k=n$, we find that the pdf associated with the survival function of Equation 6 is given by

$$\begin{aligned}f_{\boldsymbol{S}}\left(s_1,\ldots,s_n;\boldsymbol{\mu},\boldsymbol{\sigma},\beta\right) &= \frac{\partial^n}{\partial s_1\cdots\partial s_n}F_{\boldsymbol{S}}(s_1,\ldots,s_n;\boldsymbol{\mu},\boldsymbol{\sigma},\beta)\\ &= (-1)^n\bar{F}_{\boldsymbol{S}}^{(n)}\left(s_1,\ldots,s_n;\boldsymbol{\mu},\boldsymbol{\sigma},\beta\right)\\ &= \left(\prod_{i=1}^{n}\frac{\beta\cdot i}{\sigma_i}\left(\frac{s_i-\mu_i}{\sigma_i}\right)^{\beta-1}\right)\left[1+\sum_{i=1}^{n}\left(\frac{s_i-\mu_i}{\sigma_i}\right)^{\beta}\right]^{-(n+1)}\\ &= \beta^n\frac{n!\prod_{i=1}^{n}\frac{1}{\sigma_i}\left(\frac{s_i-\mu_i}{\sigma_i}\right)^{\beta-1}}{\left[1+\sum_{i=1}^{n}\left(\frac{s_i-\mu_i}{\sigma_i}\right)^{\beta}\right]^{n+1}}, \qquad \boldsymbol{s}>\boldsymbol{\mu},\end{aligned}$$

which indeed coincides with Equation 7.

Derivation of the Marginal Distribution (Equation 8). The survival function of the marginal distribution is

$$\begin{aligned}\bar{F}_{S_i}(s_i;\mu_i,\sigma_i,\beta) &= \bar{F}_{\boldsymbol{S}}(s_1=\mu_1,\ldots,s_{i-1}=\mu_{i-1},s_i,s_{i+1}=\mu_{i+1},\ldots,s_n=\mu_n;\boldsymbol{\mu},\boldsymbol{\sigma},\beta)\\ &= \left[1+\left(\frac{s_i-\mu_i}{\sigma_i}\right)^{\beta}\right]^{-1}, \qquad s_i>\mu_i,\end{aligned}$$

so its cdf is obtained as

$$F_{S_i}\left(s_i;\mu_i,\sigma_i,\beta\right)=1-\bar{F}_{S_i}\left(s_i;\mu_i,\sigma_i,\beta\right)=1-\frac{1}{1+\left(\frac{s_i-\mu_i}{\sigma_i}\right)^{\beta}}=\frac{\left(\frac{s_i-\mu_i}{\sigma_i}\right)^{\beta}}{1+\left(\frac{s_i-\mu_i}{\sigma_i}\right)^{\beta}}=\frac{1}{1+\left(\frac{s_i-\mu_i}{\sigma_i}\right)^{-\beta}}, \tag{8}$$

and its pdf is

$$f_{S_i}\left(s_i;\mu_i,\sigma_i,\beta\right)=\beta\frac{\frac{1}{\sigma_i}\left(\frac{s_i-\mu_i}{\sigma_i}\right)^{\beta-1}}{\left(1+\left(\frac{s_i-\mu_i}{\sigma_i}\right)^{\beta}\right)^{2}},$$

which is a univariate Pareto type III distribution. Its mode, for $\beta>1$, is (7, Eq. 3.3.4)

$$\mu_i+\sigma_i\left(\frac{\beta-1}{\beta+1}\right)^{1/\beta}.$$

Derivation of Moments (Including Equations 9 and 11). From (7, Eq. 6.1.27) it follows,[§] with $\hat{\alpha}=1$ and $\gamma_i=1/\beta$ and using $\Gamma(1)=1$, that for $\beta>1$ the mean is given by

$$\mathbb{E}[S_i]=\mu_i+\sigma_i\Gamma\left(\frac{\beta-1}{\beta}\right)\Gamma\left(\frac{\beta+1}{\beta}\right)=\mu_i+\sigma_i\frac{\pi/\beta}{\sin(\pi/\beta)}, \tag{9}$$

where Γ is the Gamma function and the second equality follows from its recursive expression and Euler's reflection formula, which implies that

$$\Gamma(1+z)\Gamma(1-z)=z\Gamma(z)\Gamma(1-z)=z\frac{\pi}{\sin(\pi z)},$$

for all $z\notin\mathbb{Z}$. From (7, Eq. 6.1.22) it follows that the conditional mean, for $\beta>1/n$, is given by

$$\mathbb{E}[S_i|\{S_j=s_j\}_{j\neq i}]=\mu_i+\sigma_i\left[1+\sum_{j\neq i}\left(\frac{s_j-\mu_j}{\sigma_j}\right)^{\beta}\right]^{1/\beta}\frac{\Gamma\left(n-1/\beta\right)\Gamma\left(\frac{\beta+1}{\beta}\right)}{\Gamma(n)}.$$

[§] We write $\hat{\alpha}$ for the α in (7), to differentiate it from the exponent in the divisive normalization transform.

From (7, Eq. 3.3.11) it follows that, for $\beta > 2$, the variance of the Pareto type III distribution is[¶]

$$\mathrm{Var}(S_i) = \sigma_i^2 \left[\Gamma\left(\frac{\beta-2}{\beta}\right)\Gamma\left(\frac{\beta+2}{\beta}\right) - \left(\Gamma\left(\frac{\beta-1}{\beta}\right)\Gamma\left(\frac{\beta+1}{\beta}\right)\right)^2 \right] = \sigma_i^2 \left[\frac{2\pi/\beta}{\sin(2\pi/\beta)} - \left(\frac{\pi/\beta}{\sin(\pi/\beta)}\right)^2 \right].$$

The covariance (Equation 11) is obtained (7, Eq. 6.1.29), for $\beta > 2$, as

$$\mathrm{Cov}(S_i, S_j) = \sigma_i\sigma_j \left(\Gamma\left(\frac{\beta+1}{\beta}\right)\right)^2 \left(\Gamma\left(\frac{\beta-2}{\beta}\right) - \left(\Gamma\left(\frac{\beta-1}{\beta}\right)\right)^2\right), \qquad i \neq j, \qquad [11]$$

so that the correlation coefficient is

$$\mathrm{Corr}(S_i, S_j) = \frac{\mathrm{Cov}(S_i, S_j)}{\sqrt{\mathrm{Var}(S_i)\mathrm{Var}(S_j)}} = \frac{\Gamma\left(\frac{\beta-2}{\beta}\right) - \left(\Gamma\left(\frac{\beta-1}{\beta}\right)\right)^2}{\frac{\Gamma\left(\frac{\beta+2}{\beta}\right)}{\left(\Gamma\left(\frac{\beta+1}{\beta}\right)\right)^2}\Gamma\left(\frac{\beta-2}{\beta}\right) - \left(\Gamma\left(\frac{\beta-1}{\beta}\right)\right)^2}, \qquad i \neq j.$$

Derivation of the Conditional Variance for $\beta = 1$ (Equation 12). The variance of a univariate Pareto type II distribution with cumulative distribution function (7, Eq. 3.2.2)

$$P^{(II)}(x; \mu, \sigma, \hat{\alpha}) = 1 - \left[1 + \frac{x-\mu}{\sigma}\right]^{-\hat{\alpha}} \qquad x > \mu$$

is, for any $\hat{\alpha} > 2$, given by (7, Eq. 3.3.13)

$$\mathrm{Var}(X) = \sigma^2 \left[\frac{\Gamma(\hat{\alpha}-2)\Gamma(3)}{\Gamma(\hat{\alpha})} - \left(\frac{\Gamma(\hat{\alpha}-1)\Gamma(2)}{\Gamma(\hat{\alpha})}\right)^2\right] = \sigma^2 \left[\frac{\Gamma(3)}{(\hat{\alpha}-1)(\hat{\alpha}-2)} - \frac{\Gamma(2)^2}{(\hat{\alpha}-1)^2}\right] = \sigma^2 \frac{\hat{\alpha}}{(\hat{\alpha}-1)^2(\hat{\alpha}-2)}.$$

From Equation 13 (see below) we know that the conditional distribution of a multivariate Pareto III distribution with $\beta = 1$ is a univariate Pareto type II distribution with location $\mu = \mu_i$, scale $\sigma = \sigma_i \left[1 + \sum_{j\neq i} \left(\frac{s_j - \mu_j}{\sigma_j}\right)\right]$, and shape parameter $\hat{\alpha} = n$. For any $n > 2$ we thus have

$$\begin{aligned}\mathrm{Var}(S_i | \{S_j = s_j\}_{j\neq i}) &= \sigma_i^2 \frac{n}{(n-1)^2(n-2)} \left[1 + \sum_{j\neq i} \left(\frac{s_j - \mu_j}{\sigma_j}\right)\right]^2 \\ &= \sigma_i^2 \frac{n}{(n-1)^2(n-2)} \left[1 + 2\sum_{j\neq i} \frac{s_j-\mu_j}{\sigma_j} + \sum_{j\neq i}\sum_{k\neq i, k\neq j} \frac{s_j-\mu_j}{\sigma_j}\frac{s_k-\mu_k}{\sigma_k} + \sum_{j\neq i}\left(\frac{s_j-\mu_j}{\sigma_j}\right)^2\right],\end{aligned} \qquad [12]$$

where we have used

$$\left(1 + \sum_{j=1}^{n-1} a_j\right)^2 = 1 + 2\sum_{j=1}^{n-1} a_j + \left(\sum_{j=1}^{n-1} a_j\right)^2 = 1 + 2\sum_{j=1}^{n-1} a_j + \sum_{j=1}^{n-1}\sum_{k=1,k\neq j}^{n-1} a_j a_k + \sum_{j=1}^{n-1} a_j^2.$$

Derivation of the Conditional Distribution (Equation 13). From (7, Eq. 6.1.20), with $\hat{\alpha} = 1$ and $\gamma_i = 1/\beta$, it follows that the conditional distribution of the multivariate Pareto type III distribution of Equation 6 is a univariate Pareto type IV distribution,

$$S_i | \{S_j = s_j\}_{j\neq i} \sim P^{(IV)}\left(s_i; \mu = \mu_i, \sigma = \sigma_i \left[1 + \sum_{j\neq i}\left(\frac{s_j-\mu_j}{\sigma_j}\right)^\beta\right]^{1/\beta}, \gamma = 1/\beta, \hat{\alpha} = 1 + n - 1\right),$$

where the cdf of a univariate Pareto type IV distribution is (7, Eq. 3.2.8)

$$P^{(IV)}(x; \mu, \sigma, \gamma, \hat{\alpha}) = 1 - \left[1 + \left(\frac{x-\mu}{\sigma}\right)^{1/\gamma}\right]^{-\hat{\alpha}} \qquad x > \mu.$$

This implies that the conditional distribution has cdf, for $s_i > \mu_i$,

$$F_{S_i|\{S_j=s_j\}_{j\neq i}}(s_i; \{s_j\}_{j\neq i}, \boldsymbol{\mu}, \boldsymbol{\sigma}, \beta) = 1 - \left[1 + \left(\frac{s_i - \mu_i}{\sigma_i\left[1 + \sum_{j\neq i}\left(\frac{s_j-\mu_j}{\sigma_j}\right)^\beta\right]^{1/\beta}}\right)^\beta\right]^{-n} = 1 - \left[1 + \frac{\left(\frac{s_i-\mu_i}{\sigma_i}\right)^\beta}{1 + \sum_{j\neq i}\left(\frac{s_j-\mu_j}{\sigma_j}\right)^\beta}\right]^{-n}. \qquad [13]$$

[¶] A minus sign appears to be missing in (7, Eq. 6.1.28).

Proof of Proposition 4. We have $U_i \overset{iid}{\sim} \text{Exp}(\lambda = 1)$ with c.d.f. $P(U_i \leq u_i) = 1 - \exp(-u_i)$ and, independently of all U_i, $Z \sim \text{Exp}(\lambda = 1)$ with p.d.f. $f_Z(z) = \exp(-z)$. This implies

$$\begin{aligned}
P(S_1 > s_1, \ldots, S_n > s_n) &= P\left(U_1 > Z\left(\frac{s_1 - \mu_1}{\sigma_1}\right)^\beta, \ldots, U_n > Z\left(\frac{s_n - \mu_n}{\sigma_n}\right)^\beta\right) \\
&= \int_0^\infty P\left(U_1 > Z\left(\frac{s_1 - \mu_1}{\sigma_1}\right)^\beta, \ldots, U_n > Z\left(\frac{s_n - \mu_n}{\sigma_n}\right)^\beta \middle| Z = z\right) \exp(-z) dz \\
&= \int_0^\infty \exp(-z) \prod_{i=1}^n P\left(U_i > z\left(\frac{s_i - \mu_i}{\sigma_i}\right)^\beta\right) dz \\
&= \int_0^\infty \exp(-z) \prod_{i=1}^n \exp\left(-z\left(\frac{s_i - \mu_i}{\sigma_i}\right)^\beta\right) dz \\
&= \int_0^\infty \exp\left(-z\left[1 + \sum_{i=1}^n \left(\frac{s_i - \mu_i}{\sigma_i}\right)^\beta\right]\right) dz \\
&= -\left[1 + \sum_{i=1}^n \left(\frac{s_i - \mu_i}{\sigma_i}\right)^\beta\right]^{-1} \left[\exp\left(-z\left[1 + \sum_{i=1}^n \left(\frac{s_i - \mu_i}{\sigma_i}\right)^\beta\right]\right)\right]_0^\infty \\
&= \left[1 + \sum_{i=1}^n \left(\frac{s_i - \mu_i}{\sigma_i}\right)^\beta\right]^{-1},
\end{aligned}$$

where the third equality follows from the independence of Z and all U_i.

References

1. DA Harville, *Matrix algrebra from a statistician's perspective.* (Springer-Verlag, New York), (2008).
2. TM Cover, JA Thomas, *Elements of information theory.* (Wiley-Interscience, Hoboken, NJ), (2006).
3. JP Nadal, N Parga, Nonlinear neurons in the low-noise limit: a factorial code maximizes information transfer. *Network: Comput. Neural Syst.* **5**, 565–581 (1994).
4. AJ Bell, TJ Sejnowski, An Information-Maximization Approach to Blind Separation and Blind Deconvolution. *Neural Comput.* **7**, 1129–1159 (1995).
5. JK Blitzstein, J Hwang, *Introduction to Probability.* (Chapman and Hall/CRC), 2nd edition, (2019).
6. RL Schilling, *Measures, Integrals and Martingales.* (Cambridge University Press), (2005).
7. BC Arnold, *Pareto Distributions.* (Chapman and Hall/CRC, Boca Raton), 2nd edition, (2015).

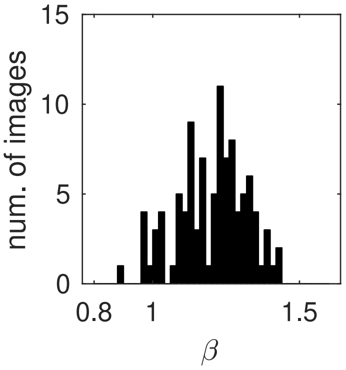

Fig. S1. Histogram of estimates of the Pareto distribution's parameter β across images, from the empirical analysis of filter responses to naturalistic images.

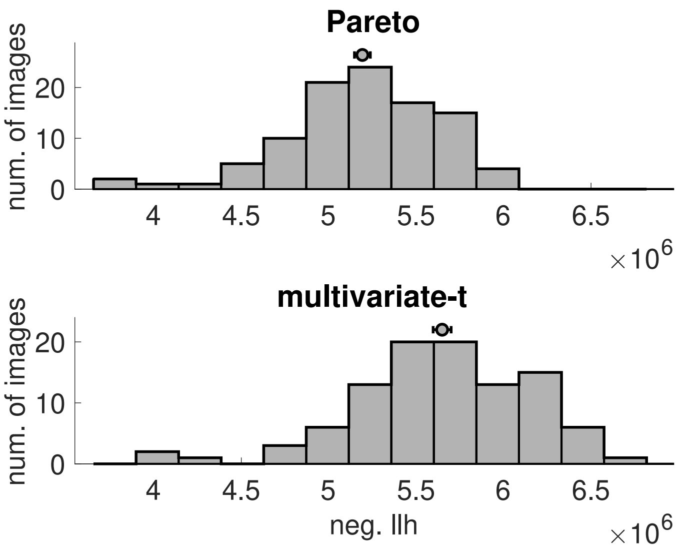

Fig. S2. Histograms showing the negative log-likelihood of the best-fitting Pareto (top) and multivariate-t (bottom) models to the statistics of naturalistic images. The counts on the y-axis correspond to the number of images, error bars show the standard error of the mean. The figure is analogous to Figure 3A in the main text but shows *negative* log-likelihoods, so that the goodness of fit is decreasing along the x-axis.

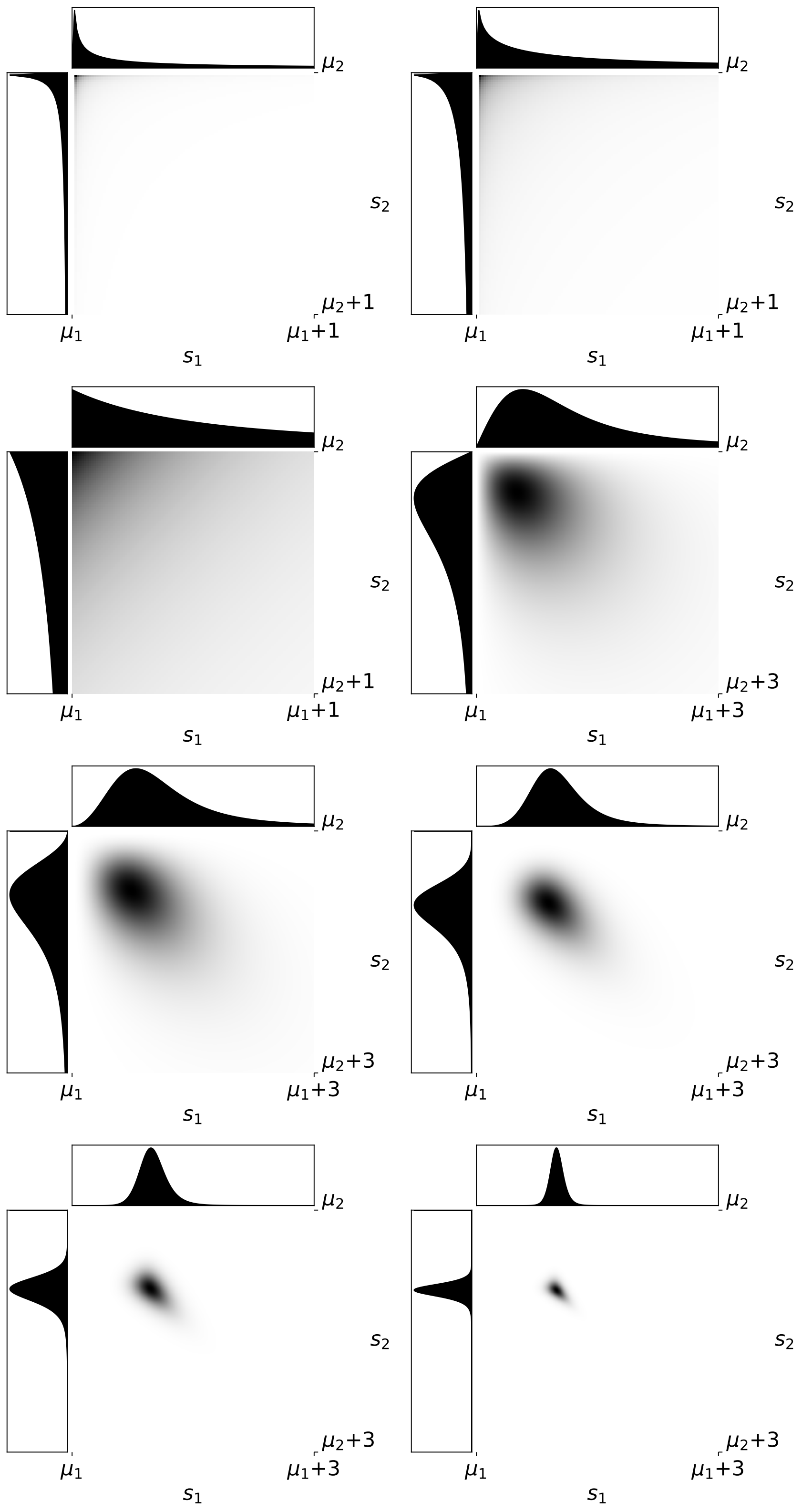

Fig. S3. Joint density of the Pareto distribution for $n = 2$ and a range of values of β. Row-wise from left to right: $\beta = 0.5, 0.75, 1, 2, 3, 5, 10, 20$.

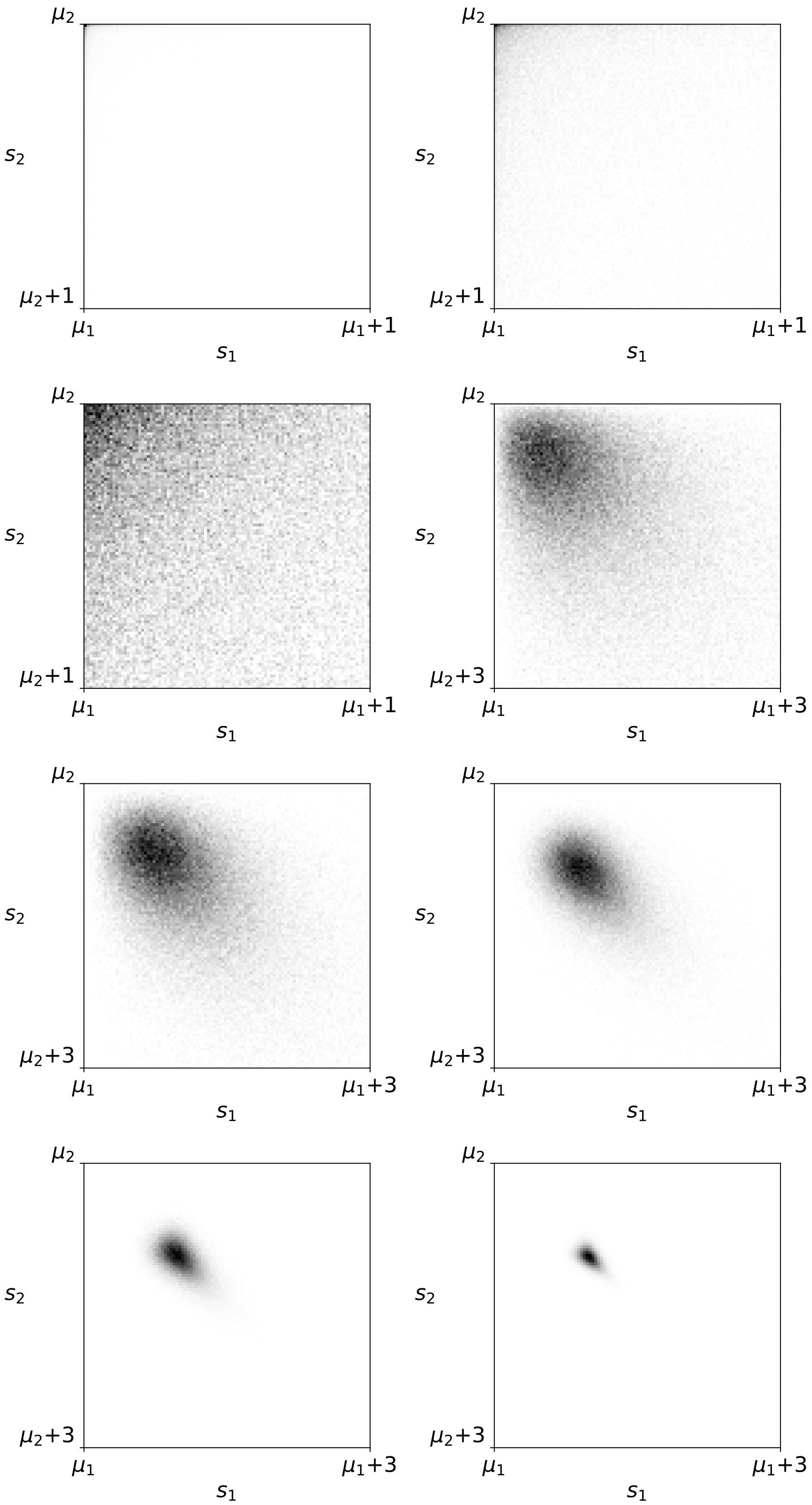

Fig. S4. Joint histogram of 100000 draws of the Pareto distribution with $n = 2$ for a range of values of β. Row-wise from left to right: $\beta = 0.5, 0.75, 1, 2, 3, 5, 10, 20.$

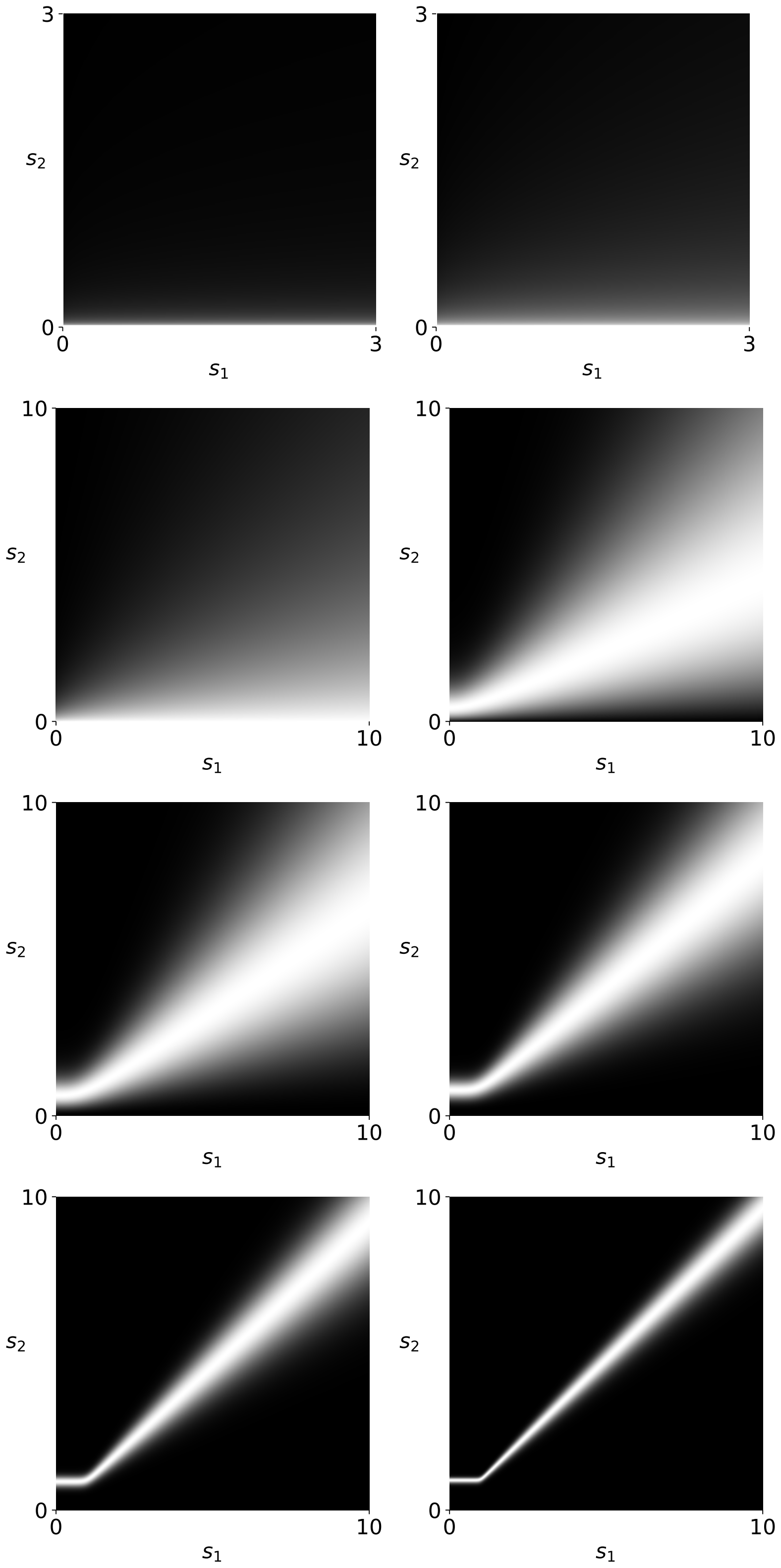

Fig. S5. Conditional density where $\boldsymbol{s}$ follows a bivariate Pareto distribution with $\boldsymbol{\mu} = \mathbf{0}$, $\boldsymbol{\sigma} = \mathbf{1}$, and a range of values of β. Row-wise from left to right: $\beta = 0.5, 0.75, 1, 2, 3, 5, 10, 20$.

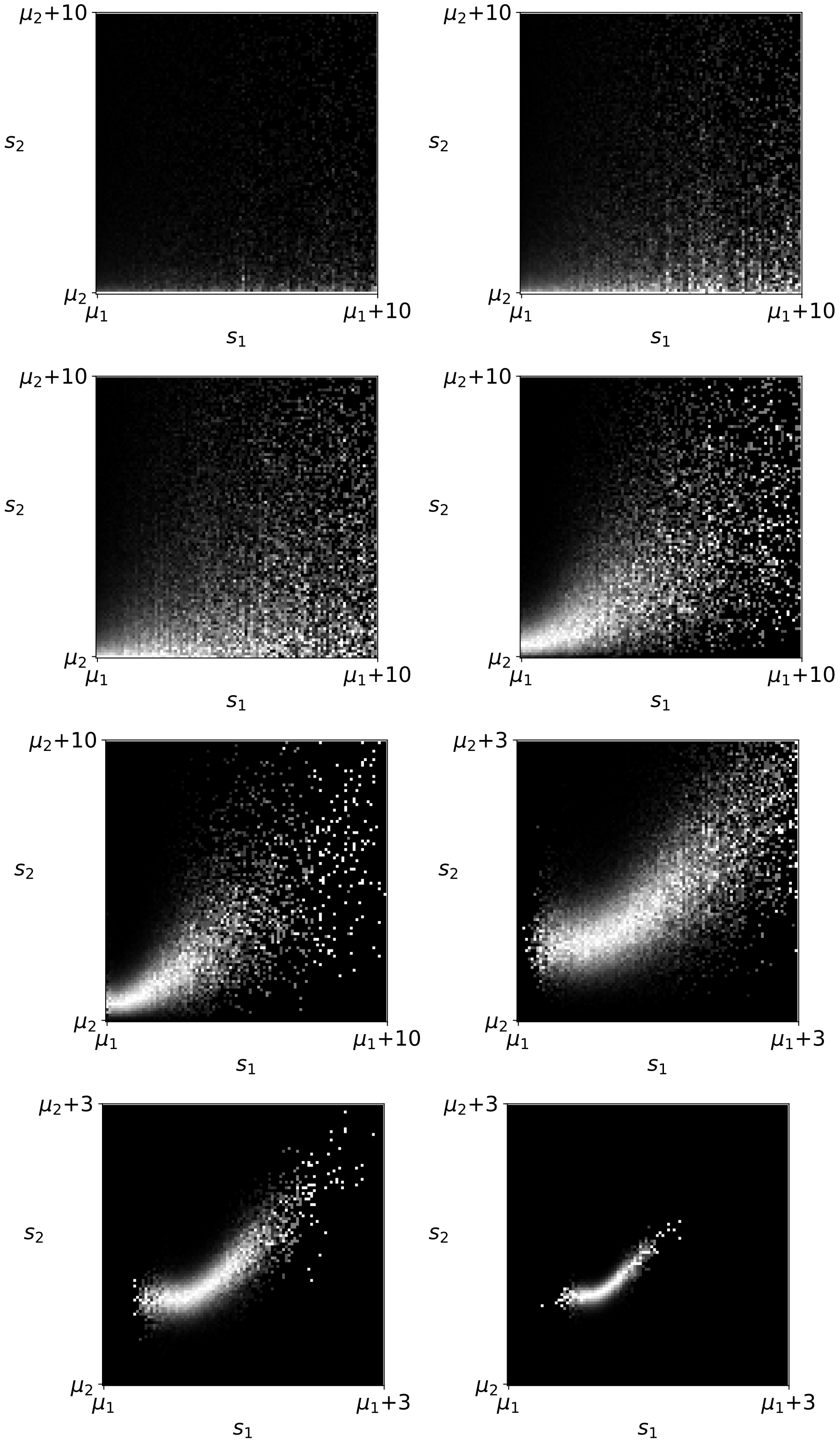

Fig. S6. Conditional histogram where $\boldsymbol{s}$ follows a bivariate Pareto distribution with $\boldsymbol{\mu} = \boldsymbol{0}$, $\boldsymbol{\sigma} = \boldsymbol{1}$, and a range of values of β. Row-wise from left to right: $\beta = 0.5, 0.75, 1, 2, 3, 5, 10, 20$.

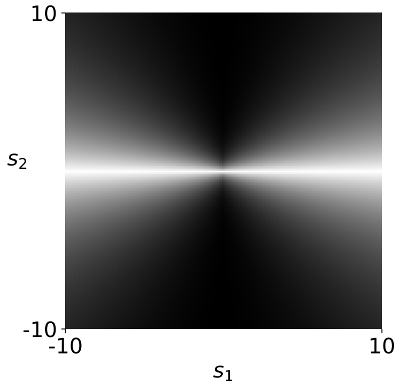

Fig. S7. Conditional density of a bivariate Pareto distribution extended to $\mathbb{R}^2$, with $\boldsymbol{\mu} = \mathbf{0}$, $\boldsymbol{\sigma} = \mathbf{1}$, and $\beta = 1$.

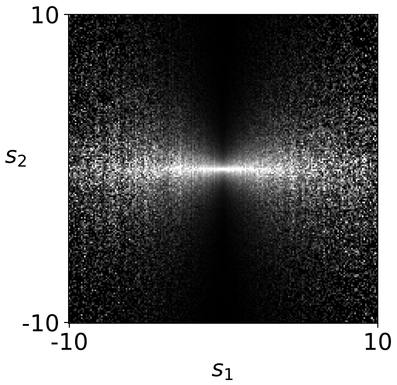

Fig. S8. Conditional histogram of a bivariate Pareto distribution extended to $\mathbb{R}^2$, with $\boldsymbol{\mu} = \mathbf{0}$, $\boldsymbol{\sigma} = \mathbf{1}$, and $\beta = 1$.

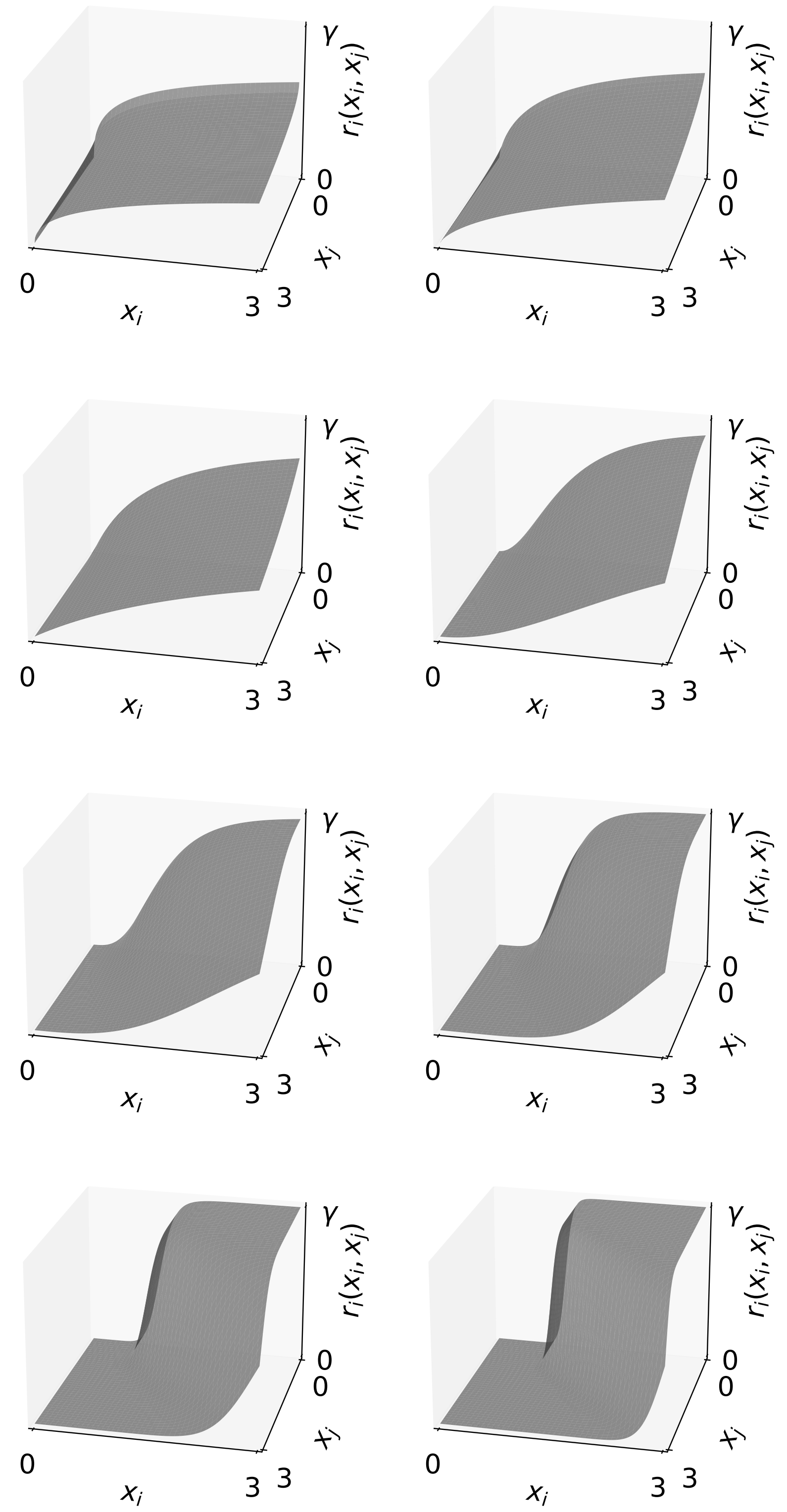

Fig. S9. Divisive normalization transform for $n = 2$ and a range of values of α. Row-wise from left to right: $\alpha = 0.5, 0.75, 1, 2, 3, 5, 10, 20$.
